# Supplementary material for: Systemic evaluation and localization of resistin expression in normal human tissues by a newly developed monoclonal antibody
Source: PLoS One. 2020 Jul 1;15(7):e0235546. doi: 10.1371/journal.pone.0235546 (PMC7329134; doi:10.1371/journal.pone.0235546)
Supplement: S1 Table — (PDF) [file pone.0235546.s001.pdf]

S1 Table. Immunopathology Evaluation: Cross Reactivity of hResistin IgG with Normal Human Tissues

Tissue	Source	Run	Test Article (hResistin IgG)		Control Article (HuIgG1)		Assay Control	Tissue Validation (Tissue Staining) Control	Tissue Comments/Nonspecific Findings
			20 µg/mL	5 µg/mL	20 µg/mL	5 µg/mL			
**Positive Control Material**									
Recombinant human resistin-FLAG UV-resin spot slides	rhResistin-FLAG	1,2,3,5	1-3+	1-3+	Neg	Neg	Neg	NS	Weak to strong staining of proteinaceous material coincident with spotted recombinant human resistin (hResistin)-FLAG with both concentrations of hResistin IgG. No staining of positive control material with either concentration of human (Hu) IgG1 or in the assay control slide.
**Negative Control Material**									
Human hypercalcemia of malignancy peptide, amino acid residues 1-34, UV-resin spot slides	PTHrP 1-34	1,2,3,5	Neg	Neg	Neg	Neg	Neg	NS	No staining of negative control PTHrP 1-34 protein spots with either concentration of hResistin IgG or HuIgG1 or in the assay control slide. Incomplete washing of slide 1 in runs 2 and 3; did not preclude interpretation.

± = equivocal, 1+ = weak, 2+ = moderate, 3+ = strong, 4+ = intense, Neg = Negative, Pos = Positive, M = Missing, NE = Not Evaluated, NS = Not Stained, freq = frequent, occas = occasional. Frequency modifiers were included to provide the approximate percentage staining of expected numbers of that cell type or tissue element at that location. The frequency of cells with staining was identified as follows: very rare (<1% of cells of a particular cell type); rare (1-5% of cells of a particular cell type); rare to occasional (>5-25% of cells of a particular cell type); occasional (>25-50% of cells of a particular type); occasional to frequent (>50-75% of cells of a particular cell type); frequent (>75-100% of cells of a particular cell type).

S1 Table. Immunopathology Evaluation: Cross Reactivity of hResistin IgG with Normal Human Tissues

Tissue	Source	Run	Test Article (hResistin IgG)		Control Article (HuIgG1)		Assay Control	Tissue Validation (Tissue Staining) Control	Tissue Comments/Nonspecific Findings
			20 µg/mL	5 µg/mL	20 µg/mL	5 µg/mL			
									General Comments: All tissue specimens judged adequate for interpretation unless otherwise specified. Background staining due to incompletely quenched endogenous myeloperoxidase or endogenous/exogenous pigments described for individual tissues.<br><br>Nuclear staining of multiple cell types was observed in the majority of tissues. This staining was sometimes quite intense in slide 1 and reduced in intensity and frequency in slide 2. Nuclear staining was judged nonspecific and hampered but did not preclude interpretation.<br><br>Slides were numbered according to the following scheme:<br>Slide 1 (hResistin IgG, 20 µg/mL),<br>Slide 2 (hResistin IgG, 5 µg/mL),<br>Slide 3 (HuIgG1, 20 µg/mL),<br>Slide 4 (HuIgG1, 5 µg/mL),<br>Slide 5 (Assay Control [omit primary antibody]), Slide 6 (Anti-β2-microglobulin [tissue staining control]).

± = equivocal, 1+ = weak, 2+ = moderate, 3+ = strong, 4+ = intense, Neg = Negative, Pos = Positive, M = Missing, NE = Not Evaluated, NS = Not Stained, freq = frequent, occas = occasional. Frequency modifiers were included to provide the approximate percentage staining of expected numbers of that cell type or tissue element at that location. The frequency of cells with staining was identified as follows: very rare (<1% of cells of a particular cell type); rare (1-5% of cells of a particular cell type); rare to occasional (>5-25% of cells of a particular cell type); occasional (>25-50% of cells of a particular type); occasional to frequent (>50-75% of cells of a particular cell type); frequent (>75-100% of cells of a particular cell type).

S1 Table. Immunopathology Evaluation: Cross Reactivity of hResistin IgG with Normal Human Tissues

Tissue	Source	Run	Test Article (hResistin IgG) 20 µg/mL	Test Article (hResistin IgG) 5 µg/mL	Control Article (HuIgG1) 20 µg/mL	Control Article (HuIgG1) 5 µg/mL	Assay Control	Tissue Validation (Tissue Staining) Control	Tissue Comments/Nonspecific Findings
**Adrenal**	****50-2	1,4						Pos	Nonspecific staining of pigment granules in slides 1 and 2.
Extracellular material			1-3+ (occas to freq)	1-3+ (occas to freq)	Neg	Neg	Neg		Observed in interstitium/stroma particularly in perivascular areas. Staining often appeared as diffuse to finely punctate material and often lined up on extracellular matrix fibers.
Macrophages (cytoplasmic granules)			1-3+ (very rare)	1-3+ (very rare)	Neg	Neg	Neg		Observed scattered in interstitium.
Other elements			Neg	Neg	Neg	Neg	Neg		
**Adrenal**	****51-2	1,4						Pos	Nonspecific staining of pigment granules in slides 1 and 2.
Extracellular material			1-3+ (occas to freq)	1-3+ (occas to freq)	Neg	Neg	Neg		Observed in interstitium/stroma particularly in perivascular areas. Staining often appeared as diffuse to finely punctate material and often lined up on extracellular matrix fibers.
Cells/processes associated with peripheral nerves (cytoplasm)			1-3+ (rare)	1-2+ (rare)	Neg	Neg	Neg		
Other elements			Neg	Neg	Neg	Neg	Neg		
**Adrenal**	****80-1	1,4						Pos	Nonspecific staining of pigment granules in slides 1 and 2.
Extracellular material			1-3+ (occas to freq)	1-3+ (occas to freq)	Neg	Neg	Neg		Observed in interstitium/stroma particularly in perivascular areas. Staining often appeared as diffuse to finely punctate material and often lined up on extracellular matrix fibers.
Other elements			Neg	Neg	Neg	Neg	Neg		

± = equivocal, 1+ = weak, 2+ = moderate, 3+ = strong, 4+ = intense, Neg = Negative, Pos = Positive, M = Missing, NE = Not Evaluated, NS = Not Stained, freq = frequent, occas = occasional. Frequency modifiers were included to provide the approximate percentage staining of expected numbers of that cell type or tissue element at that location. The frequency of cells with staining was identified as follows: very rare (<1% of cells of a particular cell type); rare (1-5% of cells of a particular cell type); rare to occasional (>5-25% of cells of a particular cell type); occasional (>25-50% of cells of a particular type); occasional to frequent (>50-75% of cells of a particular cell type); frequent (>75-100% of cells of a particular cell type).

S1 Table. Immunopathology Evaluation: Cross Reactivity of hResistin IgG with Normal Human Tissues

Tissue	Source	Run	Test Article (hResistin IgG) 20 µg/mL	Test Article (hResistin IgG) 5 µg/mL	Control Article (HuIgG1) 20 µg/mL	Control Article (HuIgG1) 5 µg/mL	Assay Control	Tissue Validation (Tissue Staining) Control	Tissue Comments/Nonspecific Findings
**Bladder (urinary)**	****8-5	1,4						Pos	
Extracellular material			1-3+ (occas to freq)	1-3+ (occas to freq)	Neg	Neg	Neg		Observed in interstitium/stroma particularly in perivascular areas. Staining often appeared as diffuse to finely punctate material and often lined up on extracellular matrix fibers.
Macrophages (cytoplasmic granules)			1-3+ (rare)	1-3+ (rare)	Neg	Neg	Neg		Observed scattered in interstitium.
Other elements			Neg	Neg	Neg	Neg	Neg		
**Bladder (urinary)**	****44-5	1,4						Pos	
Extracellular material			1-3+ (occas to freq)	1-3+ (occas to freq)	Neg	Neg	Neg		Observed in interstitium/stroma particularly in perivascular areas. Staining often appeared as diffuse to finely punctate material and often lined up on extracellular matrix fibers.
Macrophages (cytoplasmic granules)			1-3+ (rare)	1-3+ (rare)	Neg	Neg	Neg		Observed scattered in interstitium.
Other elements			Neg	Neg	Neg	Neg	Neg		
**Bladder (urinary)**	****28-1	1,4						Pos	
Extracellular material			1-3+ (occas to freq)	1-3+ (occas to freq)	Neg	Neg	Neg		Observed in interstitium/stroma particularly in perivascular areas. Staining often appeared as diffuse to finely punctate material and often lined up on extracellular matrix fibers.
Macrophages (cytoplasmic granules)			1-3+ (rare)	1-3+ (rare)	Neg	Neg	Neg		Observed scattered in interstitium.
Other elements			Neg	Neg	Neg	Neg	Neg		

± = equivocal, 1+ = weak, 2+ = moderate, 3+ = strong, 4+ = intense, Neg = Negative, Pos = Positive, M = Missing, NE = Not Evaluated, NS = Not Stained, freq = frequent, occas = occasional. Frequency modifiers were included to provide the approximate percentage staining of expected numbers of that cell type or tissue element at that location. The frequency of cells with staining was identified as follows: very rare (<1% of cells of a particular cell type); rare (1-5% of cells of a particular cell type); rare to occasional (>5-25% of cells of a particular cell type); occasional (>25-50% of cells of a particular type); occasional to frequent (>50-75% of cells of a particular cell type); frequent (>75-100% of cells of a particular cell type).

S1 Table. Immunopathology Evaluation: Cross Reactivity of hResistin IgG with Normal Human Tissues

Tissue	Source	Run	Test Article (hResistin IgG)		Control Article (HuIgG1)		Assay Control	Tissue Validation (Tissue Staining) Control	Tissue Comments/Nonspecific Findings
			20 µg/mL	5 µg/mL	20 µg/mL	5 µg/mL			
**Blood Cells**	****51	2,4						Pos	
Extracellular material			1-2+ (freq)	1-2+ (freq)	Neg	Neg	Neg		Observed in serum.
Other elements			Neg	Neg	Neg	Neg	Neg		
**Blood Cells**	****52	2,4						Pos	
Extracellular material			1-2+ (freq)	1-2+ (freq)	Neg	Neg	Neg		Observed in serum.
Other elements			Neg	Neg	Neg	Neg	Neg		
**Blood Cells**	***22	2,4						Pos	
Extracellular material			1-2+ (freq)	1-2+ (freq)	Neg	Neg	Neg		Observed in serum.
Other elements			Neg	Neg	Neg	Neg	Neg		
**Blood Vessels (endothelium)**	All tissues	1-6	Detailed Under Individual Tissues					Pos	
**Bone Marrow**	****85-1	1,4						Pos	
Extracellular material			1-3+ (occas)	1-3+ (occas)	Neg	Neg	Neg		Observed in interstitium/stroma particularly in perivascular areas. Staining often appeared as diffuse to finely punctate material and often lined up on extracellular matrix fibers.
Hematopoietic precursor cells (cytoplasmic granules)			1-3+ (rare)	1-3+ (very rare)	Neg	Neg	Neg		
Other elements			Neg	Neg	Neg	Neg	Neg		

± = equivocal, 1+ = weak, 2+ = moderate, 3+ = strong, 4+ = intense, Neg = Negative, Pos = Positive, M = Missing, NE = Not Evaluated, NS = Not Stained, freq = frequent, occas = occasional. Frequency modifiers were included to provide the approximate percentage staining of expected numbers of that cell type or tissue element at that location. The frequency of cells with staining was identified as follows: very rare (<1% of cells of a particular cell type); rare (1-5% of cells of a particular cell type); rare to occasional (>5-25% of cells of a particular cell type); occasional (>25-50% of cells of a particular type); occasional to frequent (>50-75% of cells of a particular cell type); frequent (>75-100% of cells of a particular cell type).

S1 Table. Immunopathology Evaluation: Cross Reactivity of hResistin IgG with Normal Human Tissues

Tissue	Source	Run	Test Article (hResistin IgG)		Control Article (HuIgG1)		Assay Control	Tissue Validation (Tissue Staining) Control	Tissue Comments/Nonspecific Findings
			20 µg/mL	5 µg/mL	20 µg/mL	5 µg/mL			
**Bone Marrow**	****74-1	1,4						Pos	No tissue comments.
Hematopoietic precursor cells (cytoplasmic granules)			1-3+ (rare)	1-3+ (very rare)	Neg	Neg	Neg		
Other elements			Neg	Neg	Neg	Neg	Neg		
**Bone Marrow**	****86-1	1,4						Pos	No tissue comments.
Hematopoietic precursor cells (cytoplasmic granules)			1-3+ (rare)	1-3+ (very rare)	Neg	Neg	Neg		
Other elements			Neg	Neg	Neg	Neg	Neg		
**Brain – cerebellum**	****46-1	1,4						Pos	
Extracellular material			1-2+ (occas)	1-2+ (occas)	Neg	Neg	Neg		Observed in interstitium/stroma particularly in perivascular areas. Staining often appeared as diffuse to finely punctate material and often lined up on extracellular matrix fibers.
Axons (cytoplasm)			1-2+ (rare)	1-2+ (rare)	Neg	Neg	Neg		Primarily observed in Purkinje cell layer and molecular layer.
Other elements			Neg	Neg	Neg	Neg	Neg		
**Brain – cerebellum**	****58-1	1,4						Pos	
Extracellular material			1-2+ (occas)	1-2+ (occas)	Neg	Neg	Neg		Observed in interstitium/stroma particularly in perivascular areas. Staining often appeared as diffuse to finely punctate material and often lined up on extracellular matrix fibers.
Axons (cytoplasm)			1-2+ (rare)	1-2+ (rare)	Neg	Neg	Neg		Primarily observed in Purkinje cell layer and molecular layer.
Other elements			Neg	Neg	Neg	Neg	Neg		

± = equivocal, 1+ = weak, 2+ = moderate, 3+ = strong, 4+ = intense, Neg = Negative, Pos = Positive, M = Missing, NE = Not Evaluated, NS = Not Stained, freq = frequent, occas = occasional. Frequency modifiers were included to provide the approximate percentage staining of expected numbers of that cell type or tissue element at that location. The frequency of cells with staining was identified as follows: very rare (<1% of cells of a particular cell type); rare (1-5% of cells of a particular cell type); rare to occasional (>5-25% of cells of a particular cell type); occasional (>25-50% of cells of a particular type); occasional to frequent (>50-75% of cells of a particular cell type); frequent (>75-100% of cells of a particular cell type).

S1 Table. Immunopathology Evaluation: Cross Reactivity of hResistin IgG with Normal Human Tissues

Tissue	Source	Run	Test Article (hResistin IgG)		Control Article (HuIgG1)		Assay Control	Tissue Validation (Tissue Staining) Control	Tissue Comments/Nonspecific Findings
			20 µg/mL	5 µg/mL	20 µg/mL	5 µg/mL			
**Brain – cerebellum**	****57-1	1,4						Pos	
Extracellular material			1-2+ (occas)	1-2+ (occas)	Neg	Neg	Neg		Observed in interstitium/stroma particularly in perivascular areas. Staining often appeared as diffuse to finely punctate material and often lined up on extracellular matrix fibers.
Other elements			Neg	Neg	Neg	Neg	Neg		
**Brain – cerebral cortex**	****2-7	1,4						Pos	
Extracellular material			1-2+ (occas)	1-2+ (occas)	Neg	Neg	Neg		Observed in interstitium/stroma particularly in perivascular areas. Staining often appeared as diffuse to finely punctate material and often lined up on extracellular matrix fibers. Primarily observed in meninges.
Neuronal cell bodies/axons (cytoplasm)			1-2+ (rare)	1-2+ (rare)	Neg	Neg	Neg		
Other elements			Neg	Neg	Neg	Neg	Neg		
**Brain – cerebral cortex**	****4-1	1,4						Pos	
Extracellular material			1-2+ (occas)	1-2+ (occas)	Neg	Neg	Neg		Observed in interstitium/stroma particularly in perivascular areas. Staining often appeared as diffuse to finely punctate material and often lined up on extracellular matrix fibers. Primarily observed in meninges.
Neuronal cell bodies/axons (cytoplasm)			1-2+ (rare)	1-2+ (rare)	Neg	Neg	Neg		
Other elements			Neg	Neg	Neg	Neg	Neg		

± = equivocal, 1+ = weak, 2+ = moderate, 3+ = strong, 4+ = intense, Neg = Negative, Pos = Positive, M = Missing, NE = Not Evaluated, NS = Not Stained, freq = frequent, occas = occasional. Frequency modifiers were included to provide the approximate percentage staining of expected numbers of that cell type or tissue element at that location. The frequency of cells with staining was identified as follows: very rare (<1% of cells of a particular cell type); rare (1-5% of cells of a particular cell type); rare to occasional (>5-25% of cells of a particular cell type); occasional (>25-50% of cells of a particular type); occasional to frequent (>50-75% of cells of a particular cell type); frequent (>75-100% of cells of a particular cell type).

S1 Table. Immunopathology Evaluation: Cross Reactivity of hResistin IgG with Normal Human Tissues

Tissue	Source	Run	Test Article (hResistin IgG)		Control Article (HuIgG1)		Assay Control	Tissue Validation (Tissue Staining) Control	Tissue Comments/Nonspecific Findings
			20 µg/mL	5 µg/mL	20 µg/mL	5 µg/mL			
**Brain – cerebral cortex**	****5-1	1,4,5						Pos	No staining in slide 1 in run 1. Staining repeated in run 5 and interpretation based on evaluation of slides stained in run 5.
Extracellular material			1-2+ (occas)	1-2+ (occas)	Neg	Neg	Neg		Observed in interstitium/stroma particularly in perivascular areas. Staining often appeared as diffuse to finely punctate material and often lined up on extracellular matrix fibers. Primarily observed in meninges.
Other elements			Neg	Neg	Neg	Neg	Neg		
**Breast**	****52-1	2,4						Pos	
Extracellular material			1-2+ (occas)	1-2+ (occas)	Neg	Neg	Neg		Observed in interstitium/stroma particularly in perivascular areas. Staining often appeared as diffuse to finely punctate material and often lined up on extracellular matrix fibers.
Macrophages (cytoplasmic granules)			1-3+ (rare to occas)	1-3+ (rare to occas)	Neg	Neg	Neg		Observed scattered in interstitium.
Other elements			Neg	Neg	Neg	Neg	Neg		
**Breast**	****23-2	2,4						Pos	
Extracellular material			1-2+ (occas)	1-2+ (occas)	Neg	Neg	Neg		Observed in interstitium/stroma particularly in perivascular areas. Staining often appeared as diffuse to finely punctate material and often lined up on extracellular matrix fibers.
Macrophages (cytoplasmic granules)			1-3+ (very rare)	1-3+ (very rare)	Neg	Neg	Neg		Observed scattered in interstitium.
Other elements			Neg	Neg	Neg	Neg	Neg		

± = equivocal, 1+ = weak, 2+ = moderate, 3+ = strong, 4+ = intense, Neg = Negative, Pos = Positive, M = Missing, NE = Not Evaluated, NS = Not Stained, freq = frequent, occas = occasional. Frequency modifiers were included to provide the approximate percentage staining of expected numbers of that cell type or tissue element at that location. The frequency of cells with staining was identified as follows: very rare (<1% of cells of a particular cell type); rare (1-5% of cells of a particular cell type); rare to occasional (>5-25% of cells of a particular cell type); occasional (>25-50% of cells of a particular type); occasional to frequent (>50-75% of cells of a particular cell type); frequent (>75-100% of cells of a particular cell type).

S1 Table. Immunopathology Evaluation: Cross Reactivity of hResistin IgG with Normal Human Tissues

Tissue	Source	Run	Test Article (hResistin IgG)		Control Article (HuIgG1)		Assay Control	Tissue Validation (Tissue Staining) Control	Tissue Comments/Nonspecific Findings
			20 µg/mL	5 µg/mL	20 µg/mL	5 µg/mL			
**Breast**	****90-2	2,4						Pos	
Extracellular material			1-2+ (occas)	1-2+ (occas)	Neg	Neg	Neg		Observed in interstitium/stroma particularly in perivascular areas. Staining often appeared as diffuse to finely punctate material and often lined up on extracellular matrix fibers.
Other elements			Neg	Neg	Neg	Neg	Neg		
**Colon (large intestine)**	****58-1	1,4						Pos	
Extracellular material			1-2+ (occas to freq)	1-2+ (occas to freq)	Neg	Neg	Neg		Observed in interstitium/stroma particularly in perivascular areas. Staining often appeared as diffuse to finely punctate material and often lined up on extracellular matrix fibers.
Macrophages (cytoplasmic granules)			1-3+ (rare)	1-3+ (rare)	Neg	Neg	Neg		Observed in lamina propria and scattered in interstitium.
Other elements			Neg	Neg	Neg	Neg	Neg		
**Colon (large intestine)**	****62-1	1,4						Pos	
Extracellular material			1-3+ (occas to freq)	1-3+ (occas to freq)	Neg	Neg	Neg		Observed in interstitium/stroma particularly in perivascular areas. Staining often appeared as diffuse to finely punctate material and often lined up on extracellular matrix fibers.
Macrophages (cytoplasmic granules)			1-3+ (rare)	1-3+ (rare)	Neg	Neg	Neg		Observed in lamina propria and scattered in interstitium.
Other elements			Neg	Neg	Neg	Neg	Neg		

± = equivocal, 1+ = weak, 2+ = moderate, 3+ = strong, 4+ = intense, Neg = Negative, Pos = Positive, M = Missing, NE = Not Evaluated, NS = Not Stained, freq = frequent, occas = occasional. Frequency modifiers were included to provide the approximate percentage staining of expected numbers of that cell type or tissue element at that location. The frequency of cells with staining was identified as follows: very rare (<1% of cells of a particular cell type); rare (1-5% of cells of a particular cell type); rare to occasional (>5-25% of cells of a particular cell type); occasional (>25-50% of cells of a particular type); occasional to frequent (>50-75% of cells of a particular cell type); frequent (>75-100% of cells of a particular cell type).

S1 Table. Immunopathology Evaluation: Cross Reactivity of hResistin IgG with Normal Human Tissues

Tissue	Source	Run	Test Article (hResistin IgG) 20 µg/mL	Test Article (hResistin IgG) 5 µg/mL	Control Article (HuIgG1) 20 µg/mL	Control Article (HuIgG1) 5 µg/mL	Assay Control	Tissue Validation (Tissue Staining) Control	Tissue Comments/Nonspecific Findings
**Colon (large intestine)**	****31-2	1,4						Pos	
Extracellular material			1-2+ (occas to freq)	1-2+ (occas to freq)	Neg	Neg	Neg		Observed in interstitium/stroma particularly in perivascular areas. Staining often appeared as diffuse to finely punctate material and often lined up on extracellular matrix fibers.
Macrophages (cytoplasmic granules)			1-3+ (rare)	1-3+ (rare)	Neg	Neg	Neg		Observed in lamina propria and scattered in interstitium.
Other elements			Neg	Neg	Neg	Neg	Neg		
**Eye**	****8-6	1,4						Pos	Retina, choroid, sclera. Endogenous melanin pigment.
Extracellular material			1-2+ (occas to freq)	1-2+ (occas to freq)	Neg	Neg	Neg		Observed in interstitium/stroma particularly in perivascular areas. Staining often appeared as diffuse to finely punctate material and often lined up on extracellular matrix fibers. In the eye, this staining smeared over multiple tissue elements but was particularly prominent in sclera and retina.
Other elements			Neg	Neg	Neg	Neg	Neg		

± = equivocal, 1+ = weak, 2+ = moderate, 3+ = strong, 4+ = intense, Neg = Negative, Pos = Positive, M = Missing, NE = Not Evaluated, NS = Not Stained, freq = frequent, occas = occasional. Frequency modifiers were included to provide the approximate percentage staining of expected numbers of that cell type or tissue element at that location. The frequency of cells with staining was identified as follows: very rare (<1% of cells of a particular cell type); rare (1-5% of cells of a particular cell type); rare to occasional (>5-25% of cells of a particular cell type); occasional (>25-50% of cells of a particular type); occasional to frequent (>50-75% of cells of a particular cell type); frequent (>75-100% of cells of a particular cell type).

S1 Table. Immunopathology Evaluation: Cross Reactivity of hResistin IgG with Normal Human Tissues

Tissue	Source	Run	Test Article (hResistin IgG)		Control Article (HuIgG1)		Assay Control	Tissue Validation (Tissue Staining) Control	Tissue Comments/Nonspecific Findings
			20 µg/mL	5 µg/mL	20 µg/mL	5 µg/mL			
**Eye**	****64-4	1,4						Pos	Conjunctiva, iris, ciliary body/processes, extraocular muscle. Endogenous melanin pigment.
Extracellular material			1-2+ (occas to freq)	1-2+ (occas to freq)	Neg	Neg	Neg		Observed in interstitium/stroma particularly in perivascular areas. Staining often appeared as diffuse to finely punctate material and often lined up on extracellular matrix fibers. In the eye, this staining smeared over multiple tissue elements but was particularly prominent in sclera and ciliary body.
Macrophages (cytoplasmic granules)			1-3+ (very rare)	1-3+ (very rare)	Neg	Neg	Neg		Observed in interstitium of conjunctiva.
Other elements			Neg	Neg	Neg	Neg	Neg		
**Eye**	****83-1	1,4						Pos	Retina, choroid, sclera, iris, ciliary body/processes, cornea, conjunctiva, lens. Endogenous melanin pigment.
Extracellular material			1-2+ (occas to freq)	1-2+ (occas to freq)	Neg	Neg	Neg		Observed in interstitium/stroma particularly in perivascular areas. Staining often appeared as diffuse to finely punctate material and often lined up on extracellular matrix fibers. In the eye, this staining smeared over multiple tissue elements but was particularly prominent in sclera, retina, and ciliary body.
Macrophages (cytoplasmic granules)			1-3+ (very rare)	1-3+ (very rare)	Neg	Neg	Neg		Observed in interstitium of conjunctiva.
Other elements			Neg	Neg	Neg	Neg	Neg		

± = equivocal, 1+ = weak, 2+ = moderate, 3+ = strong, 4+ = intense, Neg = Negative, Pos = Positive, M = Missing, NE = Not Evaluated, NS = Not Stained, freq = frequent, occas = occasional. Frequency modifiers were included to provide the approximate percentage staining of expected numbers of that cell type or tissue element at that location. The frequency of cells with staining was identified as follows: very rare (<1% of cells of a particular cell type); rare (1-5% of cells of a particular cell type); rare to occasional (>5-25% of cells of a particular cell type); occasional (>25-50% of cells of a particular type); occasional to frequent (>50-75% of cells of a particular cell type); frequent (>75-100% of cells of a particular cell type).

S1 Table. Immunopathology Evaluation: Cross Reactivity of hResistin IgG with Normal Human Tissues

Tissue	Source	Run	Test Article (hResistin IgG)		Control Article (HuIgG1)		Assay Control	Tissue Validation (Tissue Staining) Control	Tissue Comments/Nonspecific Findings
			20 µg/mL	5 µg/mL	20 µg/mL	5 µg/mL			
**Fallopian Tube**	****51-1	1,4						Pos	
Extracellular material			1-3+ (occas to freq)	1-3+ (occas to freq)	Neg	Neg	Neg		Observed in interstitium/stroma particularly in perivascular areas. Staining often appeared as diffuse to finely punctate material and often lined up on extracellular matrix fibers.
Macrophages (cytoplasmic granules)			1-3+ (rare)	1-3+ (rare)	Neg	Neg	Neg		Observed scattered in interstitium.
Other elements			Neg	Neg	Neg	Neg	Neg		
**Fallopian Tube**	****58-1	1,4						Pos	
Extracellular material			1-3+ (occas to freq)	1-3+ (occas to freq)	Neg	Neg	Neg		Observed in interstitium/stroma particularly in perivascular areas. Staining often appeared as diffuse to finely punctate material and often lined up on extracellular matrix fibers.
Macrophages (cytoplasmic granules)			1-3+ (rare)	1-3+ (rare)	Neg	Neg	Neg		Observed scattered in interstitium.
Other elements			Neg	Neg	Neg	Neg	Neg		
**Fallopian Tube**	****59-1	1,4						Pos	
Extracellular material			1-3+ (occas)	1-3+ (occas)	Neg	Neg	Neg		Observed in interstitium/stroma particularly in perivascular areas. Staining often appeared as diffuse to finely punctate material and often lined up on extracellular matrix fibers.
Macrophages (cytoplasmic granules)			1-3+ (very rare)	1-3+ (very rare)	Neg	Neg	Neg		Observed scattered in interstitium.
Other elements			Neg	Neg	Neg	Neg	Neg		

± = equivocal, 1+ = weak, 2+ = moderate, 3+ = strong, 4+ = intense, Neg = Negative, Pos = Positive, M = Missing, NE = Not Evaluated, NS = Not Stained, freq = frequent, occas = occasional. Frequency modifiers were included to provide the approximate percentage staining of expected numbers of that cell type or tissue element at that location. The frequency of cells with staining was identified as follows: very rare (<1% of cells of a particular cell type); rare (1-5% of cells of a particular cell type); rare to occasional (>5-25% of cells of a particular cell type); occasional (>25-50% of cells of a particular type); occasional to frequent (>50-75% of cells of a particular cell type); frequent (>75-100% of cells of a particular cell type).

S1 Table. Immunopathology Evaluation: Cross Reactivity of hResistin IgG with Normal Human Tissues

Tissue	Source	Run	Test Article (hResistin IgG)		Control Article (HuIgG1)		Assay Control	Tissue Validation (Tissue Staining) Control	Tissue Comments/Nonspecific Findings
			20 µg/mL	5 µg/mL	20 µg/mL	5 µg/mL			
**GI Tract – esophagus**	****30-1	1,4						Pos	
Extracellular material			1-3+ (occas to freq)	1-3+ (occas to freq)	Neg	Neg	Neg		Observed in interstitium/stroma particularly in perivascular areas. Staining often appeared as diffuse to finely punctate material and often lined up on extracellular matrix fibers.
Macrophages (cytoplasmic granules)			1-3+ (rare)	1-3+ (rare)	Neg	Neg	Neg		Observed scattered in interstitium.
Other elements			Neg	Neg	Neg	Neg	Neg		
**GI Tract – esophagus**	****77-5	1,4						Pos	
Extracellular material			1-3+ (occas to freq)	1-3+ (occas to freq)	Neg	Neg	Neg		Observed in interstitium/stroma particularly in perivascular areas. Staining often appeared as diffuse to finely punctate material and often lined up on extracellular matrix fibers.
Macrophages (cytoplasmic granules)			1-3+ (rare)	1-3+ (rare)	Neg	Neg	Neg		Observed scattered in interstitium.
Other elements			Neg	Neg	Neg	Neg	Neg		
**GI Tract – esophagus**	****06-1	1,4						Pos	
Extracellular material			1-2+ (occas to freq)	1-2+ (occas to freq)	Neg	Neg	Neg		Observed in interstitium/stroma particularly in perivascular areas. Staining often appeared as diffuse to finely punctate material and often lined up on extracellular matrix fibers.
Macrophages (cytoplasmic granules)			1-3+ (rare)	1-3+ (rare)	Neg	Neg	Neg		Observed scattered in interstitium.
Other elements			Neg	Neg	Neg	Neg	Neg		

± = equivocal, 1+ = weak, 2+ = moderate, 3+ = strong, 4+ = intense, Neg = Negative, Pos = Positive, M = Missing, NE = Not Evaluated, NS = Not Stained, freq = frequent, occas = occasional. Frequency modifiers were included to provide the approximate percentage staining of expected numbers of that cell type or tissue element at that location. The frequency of cells with staining was identified as follows: very rare (<1% of cells of a particular cell type); rare (1-5% of cells of a particular cell type); rare to occasional (>5-25% of cells of a particular cell type); occasional (>25-50% of cells of a particular type); occasional to frequent (>50-75% of cells of a particular cell type); frequent (>75-100% of cells of a particular cell type).

S1 Table. Immunopathology Evaluation: Cross Reactivity of hResistin IgG with Normal Human Tissues

Tissue	Source	Run	Test Article (hResistin IgG) 20 µg/mL	Test Article (hResistin IgG) 5 µg/mL	Control Article (HuIgG1) 20 µg/mL	Control Article (HuIgG1) 5 µg/mL	Assay Control	Tissue Validation (Tissue Staining) Control	Tissue Comments/Nonspecific Findings
**GI Tract – small intestine**	****8-6	3,4						Pos	
Extracellular material			1-2+ (occas to freq)	1-2+ (occas to freq)	Neg	Neg	Neg		Observed in interstitium/stroma particularly in perivascular areas. Staining often appeared as diffuse to finely punctate material and often lined up on extracellular matrix fibers.
Macrophages (cytoplasmic granules)			1-3+ (rare)	1-3+ (rare)	Neg	Neg	Neg		Observed scattered in interstitium.
Cells/processes associated with peripheral nerves (cytoplasm)			1-2+ (occas)	1-2+ (occas)	Neg	Neg	Neg		Includes myenteric plexus.
Other elements			Neg	Neg	Neg	Neg	Neg		
**GI Tract – small intestine**	****31-1	3,4						Pos	
Extracellular material			1-2+ (occas to freq)	1-2+ (occas to freq)	Neg	Neg	Neg		Observed in interstitium/stroma particularly in perivascular areas. Staining often appeared as diffuse to finely punctate material and often lined up on extracellular matrix fibers.
Macrophages (cytoplasmic granules)			1-3+ (rare)	1-3+ (rare)	Neg	Neg	Neg		Observed scattered in interstitium.
Cells/processes associated with peripheral nerves (cytoplasm)			1-2+ (occas)	1-2+ (occas)	Neg	Neg	Neg		Includes myenteric plexus.
Other elements			Neg	Neg	Neg	Neg	Neg		
**GI Tract – small intestine**	****81-1	3,4						Pos	
Extracellular material			1-2+ (occas to freq)	1-2+ (occas to freq)	Neg	Neg	Neg		Observed in interstitium/stroma particularly in perivascular areas. Staining often appeared as diffuse to finely punctate material and often lined up on extracellular matrix fibers.

± = equivocal, 1+ = weak, 2+ = moderate, 3+ = strong, 4+ = intense, Neg = Negative, Pos = Positive, M = Missing, NE = Not Evaluated, NS = Not Stained, freq = frequent, occas = occasional. Frequency modifiers were included to provide the approximate percentage staining of expected numbers of that cell type or tissue element at that location. The frequency of cells with staining was identified as follows: very rare (<1% of cells of a particular cell type); rare (1-5% of cells of a particular cell type); rare to occasional (>5-25% of cells of a particular cell type); occasional (>25-50% of cells of a particular type); occasional to frequent (>50-75% of cells of a particular cell type); frequent (>75-100% of cells of a particular cell type).

S1 Table. Immunopathology Evaluation: Cross Reactivity of hResistin IgG with Normal Human Tissues

Tissue	Source	Run	Test Article (hResistin IgG) 20 µg/mL	Test Article (hResistin IgG) 5 µg/mL	Control Article (HuIgG1) 20 µg/mL	Control Article (HuIgG1) 5 µg/mL	Assay Control	Tissue Validation (Tissue Staining) Control	Tissue Comments/Nonspecific Findings
Macrophages (cytoplasmic granules)			1-3+ (rare)	1-3+ (rare)	Neg	Neg	Neg		Observed scattered in interstitium.
Cells/processes associated with peripheral nerves (cytoplasm)			1-2+ (occas)	1-2+ (occas)	Neg	Neg	Neg		Includes myenteric plexus.
Other elements			Neg	Neg	Neg	Neg	Neg		
**GI Tract – stomach**	****46-1	3,4						Pos	
Extracellular material			1-2+ (occas to freq)	1-2+ (occas to freq)	Neg	Neg	Neg		Observed in interstitium/stroma particularly in perivascular areas. Staining often appeared as diffuse to finely punctate material and often lined up on extracellular matrix fibers.
Macrophages (cytoplasmic granules)			1-3+ (rare)	1-3+ (rare)	Neg	Neg	Neg		Observed scattered in interstitium.
Cells/processes associated with peripheral nerves (cytoplasm)			1-2+ (occas)	1-2+ (occas)	Neg	Neg	Neg		Includes myenteric plexus.
Other elements			Neg	Neg	Neg	Neg	Neg		
**GI Tract – stomach**	****56-1	3,4						Pos	
Extracellular material			1-2+ (occas to freq)	1-2+ (occas to freq)	Neg	Neg	Neg		Observed in interstitium/stroma particularly in perivascular areas. Staining often appeared as diffuse to finely punctate material and often lined up on extracellular matrix fibers.
Macrophages (cytoplasmic granules)			1-3+ (rare)	1-3+ (rare)	Neg	Neg	Neg		Observed scattered in interstitium.
Cells/processes associated with peripheral nerves (cytoplasm)			1-2+ (occas)	1-2+ (occas)	Neg	Neg	Neg		Includes myenteric plexus.
Other elements			Neg	Neg	Neg	Neg	Neg		

± = equivocal, 1+ = weak, 2+ = moderate, 3+ = strong, 4+ = intense, Neg = Negative, Pos = Positive, M = Missing, NE = Not Evaluated, NS = Not Stained, freq = frequent, occas = occasional. Frequency modifiers were included to provide the approximate percentage staining of expected numbers of that cell type or tissue element at that location. The frequency of cells with staining was identified as follows: very rare (<1% of cells of a particular cell type); rare (1-5% of cells of a particular cell type); rare to occasional (>5-25% of cells of a particular cell type); occasional (>25-50% of cells of a particular type); occasional to frequent (>50-75% of cells of a particular cell type); frequent (>75-100% of cells of a particular cell type).

S1 Table. Immunopathology Evaluation: Cross Reactivity of hResistin IgG with Normal Human Tissues

Tissue	Source	Run	Test Article (hResistin IgG) 20 µg/mL	Test Article (hResistin IgG) 5 µg/mL	Control Article (HuIgG1) 20 µg/mL	Control Article (HuIgG1) 5 µg/mL	Assay Control	Tissue Validation (Tissue Staining) Control	Tissue Comments/Nonspecific Findings
**GI Tract – stomach**	****57-1	3,4						Pos	
Extracellular material			1-2+ (occas to freq)	1-2+ (occas to freq)	Neg	Neg	Neg		Observed in interstitium/stroma particularly in perivascular areas. Staining often appeared as diffuse to finely punctate material and often lined up on extracellular matrix fibers.
Macrophages (cytoplasmic granules)			1-3+ (rare)	1-3+ (rare)	Neg	Neg	Neg		Observed scattered in interstitium.
Cells/processes associated with peripheral nerves (cytoplasm)			1-2+ (occas)	1-2+ (occas)	Neg	Neg	Neg		Includes myenteric plexus.
Other elements			Neg	Neg	Neg	Neg	Neg		
**Heart**	****82-2	1,4						Pos	
Extracellular material			1-3+ (occas)	1-3+ (occas)	Neg	Neg	Neg		Observed in interstitium/stroma particularly in perivascular areas. Staining often appeared as diffuse to finely punctate material and often lined up on extracellular matrix fibers.
Other elements			Neg	Neg	Neg	Neg	Neg		
**Heart**	****38-1	1,4						Pos	Staining of lipofuscin pigment granules in slides 1 and 2; judged nonspecific.
Extracellular material			1-3+ (occas)	1-3+ (occas)	Neg	Neg	Neg		Observed in interstitium/stroma particularly in perivascular areas. Staining often appeared as diffuse to finely punctate material and often lined up on extracellular matrix fibers.
Macrophages (cytoplasmic granules)			1-3+ (very rare)	1-3+ (very rare)	Neg	Neg	Neg		Observed scattered in interstitium.
Other elements			Neg	Neg	Neg	Neg	Neg		

± = equivocal, 1+ = weak, 2+ = moderate, 3+ = strong, 4+ = intense, Neg = Negative, Pos = Positive, M = Missing, NE = Not Evaluated, NS = Not Stained, freq = frequent, occas = occasional. Frequency modifiers were included to provide the approximate percentage staining of expected numbers of that cell type or tissue element at that location. The frequency of cells with staining was identified as follows: very rare (<1% of cells of a particular cell type); rare (1-5% of cells of a particular cell type); rare to occasional (>5-25% of cells of a particular cell type); occasional (>25-50% of cells of a particular type); occasional to frequent (>50-75% of cells of a particular cell type); frequent (>75-100% of cells of a particular cell type).

S1 Table. Immunopathology Evaluation: Cross Reactivity of hResistin IgG with Normal Human Tissues

Tissue	Source	Run	Test Article (hResistin IgG) 20 µg/mL	Test Article (hResistin IgG) 5 µg/mL	Control Article (HuIgG1) 20 µg/mL	Control Article (HuIgG1) 5 µg/mL	Assay Control	Tissue Validation (Tissue Staining) Control	Tissue Comments/Nonspecific Findings
**Heart**	****44-1	1,4						Pos	Staining of lipofuscin pigment granules in slides 1 and 2; judged nonspecific.
Extracellular material			1-3+ (occas)	1-3+ (occas)	Neg	Neg	Neg		Observed in interstitium/stroma particularly in perivascular areas. Staining often appeared as diffuse to finely punctate material and often lined up on extracellular matrix fibers.
Macrophages (cytoplasmic granules)			1-3+ (very rare)	1-3+ (very rare)	Neg	Neg	Neg		Observed scattered in interstitium.
Other elements			Neg	Neg	Neg	Neg	Neg		
**Kidney (glomerulus, tubule)**	****16-5	1,4	NE	NE	NE	NE	NE	NE	No glomeruli present. Tissue judged inadequate for evaluation and recorded as not evaluated (NE). Replaced with HT2247-1 in run 5.
**Kidney (glomerulus, tubule)**	****47-1	5,6						Pos	Replacement for HT1916-5.
Extracellular material			1-3+ (occas to freq)	1-3+ (occas to freq)	Neg	Neg	Neg		Observed in interstitium/stroma particularly in perivascular areas. Staining often appeared as diffuse to finely punctate material and often lined up on extracellular matrix fibers.
Other elements			Neg	Neg	Neg	Neg	Neg		
**Kidney (glomerulus, tubule)**	****24-1	1,4						Pos	Staining of lipofuscin granules in epithelium in slides 1 and 2; judged nonspecific.
Extracellular material			1-3+ (occas to freq)	1-3+ (occas to freq)	Neg	Neg	Neg		Observed in interstitium/stroma particularly in perivascular areas. Staining often appeared as diffuse to finely punctate material and often lined up on extracellular matrix fibers.

± = equivocal, 1+ = weak, 2+ = moderate, 3+ = strong, 4+ = intense, Neg = Negative, Pos = Positive, M = Missing, NE = Not Evaluated, NS = Not Stained, freq = frequent, occas = occasional. Frequency modifiers were included to provide the approximate percentage staining of expected numbers of that cell type or tissue element at that location. The frequency of cells with staining was identified as follows: very rare (<1% of cells of a particular cell type); rare (1-5% of cells of a particular cell type); rare to occasional (>5-25% of cells of a particular cell type); occasional (>25-50% of cells of a particular type); occasional to frequent (>50-75% of cells of a particular cell type); frequent (>75-100% of cells of a particular cell type).

S1 Table. Immunopathology Evaluation: Cross Reactivity of hResistin IgG with Normal Human Tissues

Tissue	Source	Run	Test Article (hResistin IgG)		Control Article (HuIgG1)		Assay Control	Tissue Validation (Tissue Staining) Control	Tissue Comments/Nonspecific Findings
			20 µg/mL	5 µg/mL	20 µg/mL	5 µg/mL			
Cells/processes associated with peripheral nerves (cytoplasm)			1-2+ (rare)	1-2+ (rare)	Neg	Neg	Neg		
Other elements			Neg	Neg	Neg	Neg	Neg		
**Kidney (glomerulus, tubule)**	****06-1	1,4						Pos	Staining of lipofuscin granules in epithelium in slides 1 and 2; judged nonspecific.
Extracellular material			1-3+ (occas to freq)	1-3+ (occas to freq)	Neg	Neg	Neg		Observed in interstitium/stroma particularly in perivascular areas. Staining often appeared as diffuse to finely punctate material and often lined up on extracellular matrix fibers.
Macrophages (cytoplasmic granules)			1-3+ (rare)	1-3+ (rare)	Neg	Neg	Neg		Observed scattered in interstitium.
Other elements			Neg	Neg	Neg	Neg	Neg		
**Liver**	****47-9	2,4						Pos	Endogenous bile/bilirubin pigment. Nonspecific staining of pigment granules in slides 1 and 2.
Extracellular material			1-3+ (occas)	1-3+ (occas)	Neg	Neg	Neg		Observed in interstitium/stroma particularly in perivascular areas. Staining often appeared as diffuse to finely punctate material and often lined up on extracellular matrix fibers.
Macrophages (cytoplasmic granules)			1-3+ (occas)	1-3+ (occas)	Neg	Neg	Neg		Most often observed in clusters of activated macrophages.
Other elements			Neg	Neg	Neg	Neg	Neg		

± = equivocal, 1+ = weak, 2+ = moderate, 3+ = strong, 4+ = intense, Neg = Negative, Pos = Positive, M = Missing, NE = Not Evaluated, NS = Not Stained, freq = frequent, occas = occasional. Frequency modifiers were included to provide the approximate percentage staining of expected numbers of that cell type or tissue element at that location. The frequency of cells with staining was identified as follows: very rare (<1% of cells of a particular cell type); rare (1-5% of cells of a particular cell type); rare to occasional (>5-25% of cells of a particular cell type); occasional (>25-50% of cells of a particular type); occasional to frequent (>50-75% of cells of a particular cell type); frequent (>75-100% of cells of a particular cell type).

S1 Table. Immunopathology Evaluation: Cross Reactivity of hResistin IgG with Normal Human Tissues

Tissue	Source	Run	Test Article (hResistin IgG)		Control Article (HuIgG1)		Assay Control	Tissue Validation (Tissue Staining) Control	Tissue Comments/Nonspecific Findings
			20 µg/mL	5 µg/mL	20 µg/mL	5 µg/mL			
**Liver**	****43-5	2,4						Pos	Endogenous bile/bilirubin pigment. Nonspecific staining of pigment granules in slides 1 and 2.
Extracellular material			1-3+ (occas)	1-3+ (occas)	Neg	Neg	Neg		Observed in interstitium/stroma particularly in perivascular areas. Staining often appeared as diffuse to finely punctate material and often lined up on extracellular matrix fibers.
Macrophages (cytoplasmic granules)			1-3+ (very rare)	1-3+ (very rare)	Neg	Neg	Neg		Observed scattered in interstitium.
Other elements			Neg	Neg	Neg	Neg	Neg		
**Liver**	****16-2	2,4						Pos	Endogenous bile/bilirubin pigment. Nonspecific staining of pigment granules in slides 1 and 2.
Extracellular material			1-3+ (occas)	1-3+ (occas)	Neg	Neg	Neg		Observed in interstitium/stroma particularly in perivascular areas. Staining often appeared as diffuse to finely punctate material and often lined up on extracellular matrix fibers.
Macrophages (cytoplasmic granules)			1-3+ (very rare)	1-3+ (very rare)	Neg	Neg	Neg		Observed scattered in interstitium.
Other elements			Neg	Neg	Neg	Neg	Neg		
**Lung**	****06-1	2,4						Pos	Exogenous carbon pigment.
Extracellular material			1-3+ (occas to freq)	1-3+ (occas to freq)	Neg	Neg	Neg		Observed in interstitium/stroma particularly in perivascular areas. Staining often appeared as diffuse to finely punctate material and often lined up on extracellular matrix fibers.

± = equivocal, 1+ = weak, 2+ = moderate, 3+ = strong, 4+ = intense, Neg = Negative, Pos = Positive, M = Missing, NE = Not Evaluated, NS = Not Stained, freq = frequent, occas = occasional. Frequency modifiers were included to provide the approximate percentage staining of expected numbers of that cell type or tissue element at that location. The frequency of cells with staining was identified as follows: very rare (<1% of cells of a particular cell type); rare (1-5% of cells of a particular cell type); rare to occasional (>5-25% of cells of a particular cell type); occasional (>25-50% of cells of a particular type); occasional to frequent (>50-75% of cells of a particular cell type); frequent (>75-100% of cells of a particular cell type).

S1 Table. Immunopathology Evaluation: Cross Reactivity of hResistin IgG with Normal Human Tissues

Tissue	Source	Run	Test Article (hResistin IgG) 20 µg/mL	Test Article (hResistin IgG) 5 µg/mL	Control Article (HuIgG1) 20 µg/mL	Control Article (HuIgG1) 5 µg/mL	Assay Control	Tissue Validation (Tissue Staining) Control	Tissue Comments/Nonspecific Findings
Macrophages (cytoplasmic granules)			1-3+ (very rare)	1-3+ (very rare)	Neg	Neg	Neg		Observed scattered in interstitium. Includes alveolar macrophages.
Other elements			Neg	Neg	Neg	Neg	Neg		
**Lung**	****81-1	2,4						Pos	Exogenous carbon pigment.
Extracellular material			1-3+ (occas to freq)	1-3+ (occas to freq)	Neg	Neg	Neg		Observed in interstitium/stroma particularly in perivascular areas. Staining often appeared as diffuse to finely punctate material and often lined up on extracellular matrix fibers.
Macrophages (cytoplasmic granules)			1-3+ (very rare)	1-3+ (very rare)	Neg	Neg	Neg		Observed scattered in interstitium. Includes alveolar macrophages.
Other elements			Neg	Neg	Neg	Neg	Neg		
**Lung**	****89-2	2,4						Pos	
Extracellular material			1-3+ (occas to freq)	1-3+ (occas to freq)	Neg	Neg	Neg		Observed in interstitium/stroma particularly in perivascular areas. Staining often appeared as diffuse to finely punctate material and often lined up on extracellular matrix fibers.
Macrophages (cytoplasmic granules)			1-3+ (very rare)	1-3+ (very rare)	Neg	Neg	Neg		Observed scattered in interstitium. Includes alveolar macrophages.
Other elements			Neg	Neg	Neg	Neg	Neg		
**Lymph Node**	****37-1	2,4	NE	NE	NE	NE	NE	NE	Lymph node not adequately represented in sections. Tissue recorded as not evaluated (NE). Replaced with HT2370-1 in run 5.

± = equivocal, 1+ = weak, 2+ = moderate, 3+ = strong, 4+ = intense, Neg = Negative, Pos = Positive, M = Missing, NE = Not Evaluated, NS = Not Stained, freq = frequent, occas = occasional. Frequency modifiers were included to provide the approximate percentage staining of expected numbers of that cell type or tissue element at that location. The frequency of cells with staining was identified as follows: very rare (<1% of cells of a particular cell type); rare (1-5% of cells of a particular cell type); rare to occasional (>5-25% of cells of a particular cell type); occasional (>25-50% of cells of a particular type); occasional to frequent (>50-75% of cells of a particular cell type); frequent (>75-100% of cells of a particular cell type).

S1 Table. Immunopathology Evaluation: Cross Reactivity of hResistin IgG with Normal Human Tissues

Tissue	Source	Run	Test Article (hResistin IgG)		Control Article (HuIgG1)		Assay Control	Tissue Validation (Tissue Staining) Control	Tissue Comments/Nonspecific Findings
			20 µg/mL	5 µg/mL	20 µg/mL	5 µg/mL			
**Lymph Node**	****59-1	2,4						Pos	
Extracellular material			1-3+ (occas)	1-3+ (occas)	Neg	Neg	Neg		Observed in interstitium/stroma particularly in perivascular areas. Staining often appeared as diffuse to finely punctate material and often lined up on extracellular matrix fibers.
Macrophages (cytoplasmic granules)			1-3+ (very rare)	1-3+ (very rare)	Neg	Neg	Neg		Observed scattered in interstitium.
Other elements			Neg	Neg	Neg	Neg	Neg		
**Lymph Node**	****70-1	5,6						Pos	Replacement for HT2137-1. Exogenous carbon pigment.
Extracellular material			1-3+ (occas)	1-3+ (occas)	Neg	Neg	Neg		Observed in interstitium/stroma particularly in perivascular areas. Staining often appeared as diffuse to finely punctate material and often lined up on extracellular matrix fibers.
Macrophages (cytoplasmic granules)			1-3+ (very rare)	1-3+ (very rare)	Neg	Neg	Neg		Observed scattered in interstitium.
Other elements			Neg	Neg	Neg	Neg	Neg		
**Lymph Node**	****18-2	2,4						Pos	
Extracellular material			1-3+ (occas)	1-3+ (occas)	Neg	Neg	Neg		Observed in interstitium/stroma particularly in perivascular areas. Staining often appeared as diffuse to finely punctate material and often lined up on extracellular matrix fibers.
Macrophages (cytoplasmic granules)			1-3+ (very rare)	1-3+ (very rare)	Neg	Neg	Neg		Observed scattered in interstitium.
Other elements			Neg	Neg	Neg	Neg	Neg		

± = equivocal, 1+ = weak, 2+ = moderate, 3+ = strong, 4+ = intense, Neg = Negative, Pos = Positive, M = Missing, NE = Not Evaluated, NS = Not Stained, freq = frequent, occas = occasional. Frequency modifiers were included to provide the approximate percentage staining of expected numbers of that cell type or tissue element at that location. The frequency of cells with staining was identified as follows: very rare (<1% of cells of a particular cell type); rare (1-5% of cells of a particular cell type); rare to occasional (>5-25% of cells of a particular cell type); occasional (>25-50% of cells of a particular type); occasional to frequent (>50-75% of cells of a particular cell type); frequent (>75-100% of cells of a particular cell type).

S1 Table. Immunopathology Evaluation: Cross Reactivity of hResistin IgG with Normal Human Tissues

Tissue	Source	Run	Test Article (hResistin IgG)		Control Article (HuIgG1)		Assay Control	Tissue Validation (Tissue Staining) Control	Tissue Comments/Nonspecific Findings
			20 µg/mL	5 µg/mL	20 µg/mL	5 µg/mL			
**Ovary**	****47-2	2,4						Pos	Small sample; judged adequate.
Extracellular material			1-3+ (occas to freq)	1-3+ (occas to freq)	Neg	Neg	Neg		Observed in interstitium/stroma particularly in perivascular areas. Staining often appeared as diffuse to finely punctate material and often lined up on extracellular matrix fibers.
Other elements			Neg	Neg	Neg	Neg	Neg		
**Ovary**	****51-1	2,4						Pos	
Extracellular material			1-3+ (occas to freq)	1-3+ (occas to freq)	Neg	Neg	Neg		Observed in interstitium/stroma particularly in perivascular areas. Staining often appeared as diffuse to finely punctate material and often lined up on extracellular matrix fibers.
Macrophages (cytoplasmic granules)			1-3+ (very rare)	1-3+ (very rare)	Neg	Neg	Neg		Observed scattered in interstitium.
Other elements			Neg	Neg	Neg	Neg	Neg		
**Ovary**	****22-1	2,4						Pos	
Extracellular material			1-2+ (occas)	1-2+ (occas)	Neg	Neg	Neg		Observed in interstitium/stroma particularly in perivascular areas. Staining often appeared as diffuse to finely punctate material and often lined up on extracellular matrix fibers.
Other elements			Neg	Neg	Neg	Neg	Neg		

± = equivocal, 1+ = weak, 2+ = moderate, 3+ = strong, 4+ = intense, Neg = Negative, Pos = Positive, M = Missing, NE = Not Evaluated, NS = Not Stained, freq = frequent, occas = occasional. Frequency modifiers were included to provide the approximate percentage staining of expected numbers of that cell type or tissue element at that location. The frequency of cells with staining was identified as follows: very rare (<1% of cells of a particular cell type); rare (1-5% of cells of a particular cell type); rare to occasional (>5-25% of cells of a particular cell type); occasional (>25-50% of cells of a particular type); occasional to frequent (>50-75% of cells of a particular cell type); frequent (>75-100% of cells of a particular cell type).

S1 Table. Immunopathology Evaluation: Cross Reactivity of hResistin IgG with Normal Human Tissues

Tissue	Source	Run	Test Article (hResistin IgG) 20 µg/mL	Test Article (hResistin IgG) 5 µg/mL	Control Article (HuIgG1) 20 µg/mL	Control Article (HuIgG1) 5 µg/mL	Assay Control	Tissue Validation (Tissue Staining) Control	Tissue Comments/Nonspecific Findings
**Pancreas**	****35-1	2,4						Pos	
Extracellular material			1-3+ (occas)	1-3+ (occas)	Neg	Neg	Neg		Observed in interstitium/stroma particularly in perivascular areas. Staining often appeared as diffuse to finely punctate material and often lined up on extracellular matrix fibers.
Cells/processes associated with peripheral nerves (cytoplasm)			1-3+ (occas)	1-3+ (occas)	Neg	Neg	Neg		
Macrophages (cytoplasmic granules)			1-3+ (very rare)	1-3+ (very rare)	Neg	Neg	Neg		Observed scattered in interstitium.
Other elements			Neg	Neg	Neg	Neg	Neg		
**Pancreas**	****47-1	2,4						Pos	
Extracellular material			1-3+ (occas)	1-3+ (occas)	Neg	Neg	Neg		Observed in interstitium/stroma particularly in perivascular areas. Staining often appeared as diffuse to finely punctate material and often lined up on extracellular matrix fibers.
Cells/processes associated with peripheral nerves (cytoplasm)			1-3+ (occas)	1-3+ (occas)	Neg	Neg	Neg		
Other elements			Neg	Neg	Neg	Neg	Neg		
**Pancreas**	****77-6	2,4						Pos	
Extracellular material			1-3+ (occas)	1-3+ (occas)	Neg	Neg	Neg		Observed in interstitium/stroma particularly in perivascular areas. Staining often appeared as diffuse to finely punctate material and often lined up on extracellular matrix fibers.
Cells/processes associated with peripheral nerves (cytoplasm)			1-3+ (occas)	1-3+ (occas)	Neg	Neg	Neg		

± = equivocal, 1+ = weak, 2+ = moderate, 3+ = strong, 4+ = intense, Neg = Negative, Pos = Positive, M = Missing, NE = Not Evaluated, NS = Not Stained, freq = frequent, occas = occasional. Frequency modifiers were included to provide the approximate percentage staining of expected numbers of that cell type or tissue element at that location. The frequency of cells with staining was identified as follows: very rare (<1% of cells of a particular cell type); rare (1-5% of cells of a particular cell type); rare to occasional (>5-25% of cells of a particular cell type); occasional (>25-50% of cells of a particular type); occasional to frequent (>50-75% of cells of a particular cell type); frequent (>75-100% of cells of a particular cell type).

S1 Table. Immunopathology Evaluation: Cross Reactivity of hResistin IgG with Normal Human Tissues

Tissue	Source	Run	Test Article (hResistin IgG)		Control Article (HuIgG1)		Assay Control	Tissue Validation (Tissue Staining) Control	Tissue Comments/Nonspecific Findings
			20 µg/mL	5 µg/mL	20 µg/mL	5 µg/mL			
Macrophages (cytoplasmic granules)			1-3+ (very rare)	1-3+ (very rare)	Neg	Neg	Neg		Observed scattered in interstitium.
Other elements			Neg	Neg	Neg	Neg	Neg		
**Parathyroid**	****79-1	2,4						Pos	
Extracellular material			1-2+ (occas)	1-2+ (occas)	Neg	Neg	Neg		Observed in interstitium/stroma particularly in perivascular areas. Staining often appeared as diffuse to finely punctate material.
Other elements			Neg	Neg	Neg	Neg	Neg		
**Parathyroid**	****17-2	2,4	M	M	M	M	M	M	No parathyroid present in sections. Tissue recorded as missing (M). No additional donors available within study timeframe.
**Parathyroid**	****90-1	2,4						Pos	
Extracellular material			1-2+ (occas)	1-2+ (occas)	Neg	Neg	Neg		Observed in interstitium/stroma particularly in perivascular areas. Staining often appeared as diffuse to finely punctate material.
Macrophages (cytoplasmic granules)			1-3+ (rare)	1-3+ (rare)	Neg	Neg	Neg		Observed scattered in interstitium.
Other elements			Neg	Neg	Neg	Neg	Neg		
**Peripheral Nerve**	****77-4	2,4						Pos	
Extracellular material			1-2+ (occas)	1-2+ (occas)	Neg	Neg	Neg		Observed in interstitium/stroma particularly in perivascular areas. Staining often appeared as diffuse to finely punctate material and often lined up on extracellular matrix fibers.

± = equivocal, 1+ = weak, 2+ = moderate, 3+ = strong, 4+ = intense, Neg = Negative, Pos = Positive, M = Missing, NE = Not Evaluated, NS = Not Stained, freq = frequent, occas = occasional. Frequency modifiers were included to provide the approximate percentage staining of expected numbers of that cell type or tissue element at that location. The frequency of cells with staining was identified as follows: very rare (<1% of cells of a particular cell type); rare (1-5% of cells of a particular cell type); rare to occasional (>5-25% of cells of a particular cell type); occasional (>25-50% of cells of a particular type); occasional to frequent (>50-75% of cells of a particular cell type); frequent (>75-100% of cells of a particular cell type).

S1 Table. Immunopathology Evaluation: Cross Reactivity of hResistin IgG with Normal Human Tissues

Tissue	Source	Run	Test Article (hResistin IgG)		Control Article (HuIgG1)		Assay Control	Tissue Validation (Tissue Staining) Control	Tissue Comments/Nonspecific Findings
			20 µg/mL	5 µg/mL	20 µg/mL	5 µg/mL			
Cells/processes associated with peripheral nerves (cytoplasm)			1-2+ (occas)	1-2+ (occas)	Neg	Neg	Neg		
Macrophages (cytoplasmic granules)			1-3+ (very rare)	1-3+ (very rare)	Neg	Neg	Neg		Observed scattered in interstitium.
Other elements			Neg	Neg	Neg	Neg	Neg		
**Peripheral Nerve**	****34-1	2,4						Pos	
Extracellular material			1-3+ (occas)	1-3+ (occas)	Neg	Neg	Neg		Observed in interstitium/stroma particularly in perivascular areas. Staining often appeared as diffuse to finely punctate material and often lined up on extracellular matrix fibers.
Cells/processes associated with peripheral nerves (cytoplasm)			1-3+ (occas)	1-3+ (occas)	Neg	Neg	Neg		
Macrophages (cytoplasmic granules)			1-3+ (very rare)	1-3+ (very rare)	Neg	Neg	Neg		Observed scattered in interstitium.
Other elements			Neg	Neg	Neg	Neg	Neg		
**Peripheral Nerve**	****37-4	2,4						Pos	
Extracellular material			1-2+ (occas)	1-2+ (occas)	Neg	Neg	Neg		Observed in interstitium/stroma particularly in perivascular areas. Staining often appeared as diffuse to finely punctate material and often lined up on extracellular matrix fibers.
Cells/processes associated with peripheral nerves (cytoplasm)			1-2+ (occas)	1-2+ (occas)	Neg	Neg	Neg		
Macrophages (cytoplasmic granules)			1-3+ (very rare)	1-3+ (very rare)	Neg	Neg	Neg		Observed scattered in interstitium.
Other elements			Neg	Neg	Neg	Neg	Neg		

± = equivocal, 1+ = weak, 2+ = moderate, 3+ = strong, 4+ = intense, Neg = Negative, Pos = Positive, M = Missing, NE = Not Evaluated, NS = Not Stained, freq = frequent, occas = occasional. Frequency modifiers were included to provide the approximate percentage staining of expected numbers of that cell type or tissue element at that location. The frequency of cells with staining was identified as follows: very rare (<1% of cells of a particular cell type); rare (1-5% of cells of a particular cell type); rare to occasional (>5-25% of cells of a particular cell type); occasional (>25-50% of cells of a particular type); occasional to frequent (>50-75% of cells of a particular cell type); frequent (>75-100% of cells of a particular cell type).

S1 Table. Immunopathology Evaluation: Cross Reactivity of hResistin IgG with Normal Human Tissues

Tissue	Source	Run	Test Article (hResistin IgG)		Control Article (HuIgG1)		Assay Control	Tissue Validation (Tissue Staining) Control	Tissue Comments/Nonspecific Findings
			20 µg/mL	5 µg/mL	20 µg/mL	5 µg/mL			
**Pituitary**	****18-1	2,4						Pos	Adenohypophysis.
Extracellular material			1-3+ (occas)	1-3+ (occas)	Neg	Neg	Neg		Observed in interstitium/stroma particularly in perivascular areas. Staining often appeared as diffuse to finely punctate material.
Epithelium, adenohypophysis (cytoplasm, cytoplasmic granules)			Neg	Neg	1-3+ (occas to freq)	1-3+ (occas to freq)	Neg		Judged of uncertain specificity and did not hamper interpretation of test article staining as similar staining was not observed with the test article.
Macrophages (cytoplasmic granules)			1-3+ (very rare)	1-3+ (very rare)	Neg	Neg	Neg		Observed scattered in interstitium.
Other elements			Neg	Neg	Neg	Neg	Neg		
**Pituitary**	****89-1	2,4						Pos	Adenohypophysis.
Extracellular material			1-3+ (occas)	1-3+ (occas)	Neg	Neg	Neg		Observed in interstitium/stroma particularly in perivascular areas. Staining often appeared as diffuse to finely punctate material.
Epithelium, adenohypophysis (cytoplasm, cytoplasmic granules)			Neg	Neg	1-3+ (occas to freq)	1-3+ (occas to freq)	Neg		Judged of uncertain specificity and did not hamper interpretation of test article staining as similar staining was not observed with the test article.
Macrophages (cytoplasmic granules)			1-3+ (very rare)	1-3+ (very rare)	Neg	Neg	Neg		Observed scattered in interstitium.
Other elements			Neg	Neg	Neg	Neg	Neg		

± = equivocal, 1+ = weak, 2+ = moderate, 3+ = strong, 4+ = intense, Neg = Negative, Pos = Positive, M = Missing, NE = Not Evaluated, NS = Not Stained, freq = frequent, occas = occasional. Frequency modifiers were included to provide the approximate percentage staining of expected numbers of that cell type or tissue element at that location. The frequency of cells with staining was identified as follows: very rare (<1% of cells of a particular cell type); rare (1-5% of cells of a particular cell type); rare to occasional (>5-25% of cells of a particular cell type); occasional (>25-50% of cells of a particular type); occasional to frequent (>50-75% of cells of a particular cell type); frequent (>75-100% of cells of a particular cell type).

S1 Table. Immunopathology Evaluation: Cross Reactivity of hResistin IgG with Normal Human Tissues

Tissue	Source	Run	Test Article (hResistin IgG) 20 µg/mL	Test Article (hResistin IgG) 5 µg/mL	Control Article (HuIgG1) 20 µg/mL	Control Article (HuIgG1) 5 µg/mL	Assay Control	Tissue Validation (Tissue Staining) Control	Tissue Comments/Nonspecific Findings
**Pituitary**	****83-1	5,6						Pos	Included as additional donor for representation of neurohypophysis but only adenohypophysis present.
Extracellular material			1-3+ (occas)	1-3+ (occas)	Neg	Neg	Neg		Observed in interstitium/stroma particularly in perivascular areas. Staining often appeared as diffuse to finely punctate material.
Epithelium, adenohypophysis (cytoplasm, cytoplasmic granules)			Neg	Neg	1-3+ (occas to freq)	1-3+ (occas to freq)	Neg		Judged of uncertain specificity and did not hamper interpretation of test article staining as similar staining was not observed with the test article.
Other elements			Neg	Neg	Neg	Neg	Neg		
**Pituitary**	****85-1	2,4						Pos	Adenohypophysis.
Extracellular material			1-2+ (occas)	1-2+ (occas)	Neg	Neg	Neg		Observed in interstitium/stroma particularly in perivascular areas. Staining often appeared as diffuse to finely punctate material.
Epithelium, adenohypophysis (cytoplasm, cytoplasmic granules)			Neg	Neg	1-3+ (occas)	1-3+ (occas)	Neg		Judged of uncertain specificity and did not hamper interpretation of test article staining as similar staining was not observed with the test article.
Macrophages (cytoplasmic granules)			1-3+ (rare)	1-3+ (rare)	Neg	Neg	Neg		Observed scattered in interstitium.
Other elements			Neg	Neg	Neg	Neg	Neg		

± = equivocal, 1+ = weak, 2+ = moderate, 3+ = strong, 4+ = intense, Neg = Negative, Pos = Positive, M = Missing, NE = Not Evaluated, NS = Not Stained, freq = frequent, occas = occasional. Frequency modifiers were included to provide the approximate percentage staining of expected numbers of that cell type or tissue element at that location. The frequency of cells with staining was identified as follows: very rare (<1% of cells of a particular cell type); rare (1-5% of cells of a particular cell type); rare to occasional (>5-25% of cells of a particular cell type); occasional (>25-50% of cells of a particular type); occasional to frequent (>50-75% of cells of a particular cell type); frequent (>75-100% of cells of a particular cell type).

S1 Table. Immunopathology Evaluation: Cross Reactivity of hResistin IgG with Normal Human Tissues

Tissue	Source	Run	Test Article (hResistin IgG)		Control Article (HuIgG1)		Assay Control	Tissue Validation (Tissue Staining) Control	Tissue Comments/Nonspecific Findings
			20 µg/mL	5 µg/mL	20 µg/mL	5 µg/mL			
**Pituitary**	****87-1	5,6						Pos	Included as additional donor for representation of neurohypophysis but only adenohypophysis present.
Extracellular material			1-3+ (occas to freq)	1-3+ (occas to freq)	Neg	Neg	Neg		Observed in interstitium/stroma particularly in perivascular areas. Staining often appeared as diffuse to finely punctate material and often lined up on extracellular matrix fibers.
Other elements			Neg	Neg	Neg	Neg	Neg		
**Placenta**	****21-1	2,4						Pos	
Extracellular material			1-3+ (occas to freq)	1-3+ (occas to freq)	Neg	Neg	Neg		Observed in interstitium/stroma particularly in perivascular areas. Staining often appeared as diffuse to finely punctate material and often lined up on extracellular matrix fibers.
Other elements			Neg	Neg	Neg	Neg	Neg		
**Placenta**	****74-2	2,4						Pos	
Extracellular material			1-3+ (occas to freq)	1-3+ (occas to freq)	Neg	Neg	Neg		Observed in interstitium/stroma particularly in perivascular areas. Staining often appeared as diffuse to finely punctate material and often lined up on extracellular matrix fibers.
Other elements			Neg	Neg	Neg	Neg	Neg		

± = equivocal, 1+ = weak, 2+ = moderate, 3+ = strong, 4+ = intense, Neg = Negative, Pos = Positive, M = Missing, NE = Not Evaluated, NS = Not Stained, freq = frequent, occas = occasional. Frequency modifiers were included to provide the approximate percentage staining of expected numbers of that cell type or tissue element at that location. The frequency of cells with staining was identified as follows: very rare (<1% of cells of a particular cell type); rare (1-5% of cells of a particular cell type); rare to occasional (>5-25% of cells of a particular cell type); occasional (>25-50% of cells of a particular type); occasional to frequent (>50-75% of cells of a particular cell type); frequent (>75-100% of cells of a particular cell type).

S1 Table. Immunopathology Evaluation: Cross Reactivity of hResistin IgG with Normal Human Tissues

Tissue	Source	Run	Test Article (hResistin IgG)		Control Article (HuIgG1)		Assay Control	Tissue Validation (Tissue Staining) Control	Tissue Comments/Nonspecific Findings
			20 µg/mL	5 µg/mL	20 µg/mL	5 µg/mL			
**Placenta**	****92-1	2,4						Pos	
Extracellular material			1-2+ (occas to freq)	1-2+ (occas to freq)	Neg	Neg	Neg		Observed in interstitium/stroma particularly in perivascular areas. Staining often appeared as diffuse to finely punctate material and often lined up on extracellular matrix fibers.
Other elements			Neg	Neg	Neg	Neg	Neg		
**Prostate**	****37-1	2,4						Pos	
Extracellular material			1-3+ (occas to freq)	1-3+ (occas to freq)	Neg	Neg	Neg		Observed in interstitium/stroma particularly in perivascular areas. Staining often appeared as diffuse to finely punctate material and often lined up on extracellular matrix fibers.
Macrophages (cytoplasmic granules)			1-3+ (rare)	1-3+ (rare)	Neg	Neg	Neg		Observed scattered in interstitium.
Other elements			Neg	Neg	Neg	Neg	Neg		
**Prostate**	****44-1	2,4						Pos	
Extracellular material			1-2+ (occas to freq)	1-2+ (occas to freq)	Neg	Neg	Neg		Observed in interstitium/stroma particularly in perivascular areas. Staining often appeared as diffuse to finely punctate material and often lined up on extracellular matrix fibers.
Cells/processes associated with peripheral nerves (cytoplasm)			2-3+ (rare to occas)	2-3+ (rare to occas)	Neg	Neg	Neg		
Macrophages (cytoplasmic granules)			1-3+ (rare to occas)	1-3+ (rare to occas)	Neg	Neg	Neg		Observed scattered in interstitium.
Other elements			Neg	Neg	Neg	Neg	Neg		

± = equivocal, 1+ = weak, 2+ = moderate, 3+ = strong, 4+ = intense, Neg = Negative, Pos = Positive, M = Missing, NE = Not Evaluated, NS = Not Stained, freq = frequent, occas = occasional. Frequency modifiers were included to provide the approximate percentage staining of expected numbers of that cell type or tissue element at that location. The frequency of cells with staining was identified as follows: very rare (<1% of cells of a particular cell type); rare (1-5% of cells of a particular cell type); rare to occasional (>5-25% of cells of a particular cell type); occasional (>25-50% of cells of a particular type); occasional to frequent (>50-75% of cells of a particular cell type); frequent (>75-100% of cells of a particular cell type).

S1 Table. Immunopathology Evaluation: Cross Reactivity of hResistin IgG with Normal Human Tissues

Tissue	Source	Run	Test Article (hResistin IgG) 20 µg/mL	Test Article (hResistin IgG) 5 µg/mL	Control Article (HuIgG1) 20 µg/mL	Control Article (HuIgG1) 5 µg/mL	Assay Control	Tissue Validation (Tissue Staining) Control	Tissue Comments/Nonspecific Findings
**Prostate**	****80-1	2,4						Pos	
Extracellular material			1-3+ (occas to freq)	1-3+ (occas to freq)	Neg	Neg	Neg		Observed in interstitium/stroma particularly in perivascular areas. Staining often appeared as diffuse to finely punctate material and often lined up on extracellular matrix fibers.
Cells/processes associated with peripheral nerves (cytoplasm)			2-3+ (rare to occas)	2-3+ (rare to occas)	Neg	Neg	Neg		
Macrophages (cytoplasmic granules)			1-3+ (rare)	1-3+ (rare)	Neg	Neg	Neg		Observed scattered in interstitium.
Other elements			Neg	Neg	Neg	Neg	Neg		
**Salivary Gland**	****35-2	3,4	NE	NE	NE	NE	NE	NE	Salivary gland not adequately represented in sections. Tissue recorded as not evaluated (NE). Replaced with HT2297-1 in run 5.
**Salivary Gland**	****18-2	3,4						Pos	
Extracellular material			1-3+ (occas to freq)	1-3+ (occas to freq)	Neg	Neg	Neg		Observed in interstitium/stroma particularly in perivascular areas. Staining often appeared as diffuse to finely punctate material and often lined up on extracellular matrix fibers.
Macrophages (cytoplasmic granules)			2-3+ (very rare)	2-3+ (very rare)	Neg	Neg	Neg		Observed scattered in interstitium.
Other elements			Neg	Neg	Neg	Neg	Neg		

± = equivocal, 1+ = weak, 2+ = moderate, 3+ = strong, 4+ = intense, Neg = Negative, Pos = Positive, M = Missing, NE = Not Evaluated, NS = Not Stained, freq = frequent, occas = occasional. Frequency modifiers were included to provide the approximate percentage staining of expected numbers of that cell type or tissue element at that location. The frequency of cells with staining was identified as follows: very rare (<1% of cells of a particular cell type); rare (1-5% of cells of a particular cell type); rare to occasional (>5-25% of cells of a particular cell type); occasional (>25-50% of cells of a particular type); occasional to frequent (>50-75% of cells of a particular cell type); frequent (>75-100% of cells of a particular cell type).

S1 Table. Immunopathology Evaluation: Cross Reactivity of hResistin IgG with Normal Human Tissues

Tissue	Source	Run	Test Article (hResistin IgG)		Control Article (HuIgG1)		Assay Control	Tissue Validation (Tissue Staining) Control	Tissue Comments/Nonspecific Findings
			20 µg/mL	5 µg/mL	20 µg/mL	5 µg/mL			
**Salivary Gland**	****8-8	3,4						Pos	
Extracellular material			1-3+ (occas to freq)	1-3+ (occas to freq)	Neg	Neg	Neg		Observed in interstitium/stroma particularly in perivascular areas. Staining often appeared as diffuse to finely punctate material and often lined up on extracellular matrix fibers.
Macrophages (cytoplasmic granules)			2-3+ (very rare)	2-3+ (very rare)	Neg	Neg	Neg		Observed scattered in interstitium.
Other elements			Neg	Neg	Neg	Neg	Neg		
**Salivary Gland**	****97-1	5,6						Pos	Replacement for HT1735-2.
Extracellular material			1-3+ (occas to freq)	1-3+ (occas to freq)	Neg	Neg	Neg		Observed in interstitium/stroma particularly in perivascular areas. Staining often appeared as diffuse to finely punctate material and often lined up on extracellular matrix fibers.
Other elements			Neg	Neg	Neg	Neg	Neg		
**Skin**	****48-25	3,4						Pos	
Extracellular material			1-3+ (occas to freq)	1-3+ (occas to freq)	Neg	Neg	Neg		Observed in interstitium/stroma particularly in perivascular areas. Staining often appeared as diffuse to finely punctate material and often lined up on extracellular matrix fibers.
Macrophages (cytoplasmic granules)			2-3+ (very rare)	2-3+ (very rare)	Neg	Neg	Neg		Observed scattered in interstitium.
Other elements			Neg	Neg	Neg	Neg	Neg		

± = equivocal, 1+ = weak, 2+ = moderate, 3+ = strong, 4+ = intense, Neg = Negative, Pos = Positive, M = Missing, NE = Not Evaluated, NS = Not Stained, freq = frequent, occas = occasional. Frequency modifiers were included to provide the approximate percentage staining of expected numbers of that cell type or tissue element at that location. The frequency of cells with staining was identified as follows: very rare (<1% of cells of a particular cell type); rare (1-5% of cells of a particular cell type); rare to occasional (>5-25% of cells of a particular cell type); occasional (>25-50% of cells of a particular type); occasional to frequent (>50-75% of cells of a particular cell type); frequent (>75-100% of cells of a particular cell type).

S1 Table. Immunopathology Evaluation: Cross Reactivity of hResistin IgG with Normal Human Tissues

Tissue	Source	Run	Test Article (hResistin IgG)		Control Article (HuIgG1)		Assay Control	Tissue Validation (Tissue Staining) Control	Tissue Comments/Nonspecific Findings
			20 µg/mL	5 µg/mL	20 µg/mL	5 µg/mL			
**Skin**	****96-1	3,4						Pos	
Extracellular material			1-3+ (occas)	1-3+ (occas)	Neg	Neg	Neg		Observed in interstitium/stroma particularly in perivascular areas. Staining often appeared as diffuse to finely punctate material and often lined up on extracellular matrix fibers.
Macrophages (cytoplasmic granules)			2-3+ (very rare)	2-3+ (very rare)	Neg	Neg	Neg		Observed scattered in interstitium.
Other elements			Neg	Neg	Neg	Neg	Neg		
**Skin**	****50-2	3,4						Pos	
Extracellular material			1-3+ (occas)	1-3+ (occas)	Neg	Neg	Neg		Observed in interstitium/stroma particularly in perivascular areas. Staining often appeared as diffuse to finely punctate material and often lined up on extracellular matrix fibers.
Macrophages (cytoplasmic granules)			2-3+ (very rare)	2-3+ (very rare)	Neg	Neg	Neg		Observed scattered in interstitium.
Other elements			Neg	Neg	Neg	Neg	Neg		
**Spinal Cord**	****4-4	3,4						Pos	
Extracellular material			1-2+ (occas)	1-2+ (occas)	Neg	Neg	Neg		Observed in interstitium/stroma particularly in perivascular areas. Staining often appeared as diffuse to finely punctate material and often lined up on extracellular matrix fibers. Primarily observed in meninges.
Glial cells/processes (cytoplasm)			1-2+ (occas to freq)	1-2+ (occas)	Neg	Neg	Neg		

± = equivocal, 1+ = weak, 2+ = moderate, 3+ = strong, 4+ = intense, Neg = Negative, Pos = Positive, M = Missing, NE = Not Evaluated, NS = Not Stained, freq = frequent, occas = occasional. Frequency modifiers were included to provide the approximate percentage staining of expected numbers of that cell type or tissue element at that location. The frequency of cells with staining was identified as follows: very rare (<1% of cells of a particular cell type); rare (1-5% of cells of a particular cell type); rare to occasional (>5-25% of cells of a particular cell type); occasional (>25-50% of cells of a particular type); occasional to frequent (>50-75% of cells of a particular cell type); frequent (>75-100% of cells of a particular cell type).

S1 Table. Immunopathology Evaluation: Cross Reactivity of hResistin IgG with Normal Human Tissues

Tissue	Source	Run	Test Article (hResistin IgG) 20 µg/mL	Test Article (hResistin IgG) 5 µg/mL	Control Article (HuIgG1) 20 µg/mL	Control Article (HuIgG1) 5 µg/mL	Assay Control	Tissue Validation (Tissue Staining) Control	Tissue Comments/Nonspecific Findings
Axons (cytoplasm)			2-3+ (occas to freq)	2-3+ (occas)	Neg	Neg	Neg		
Cells/processes associated with peripheral nerves (cytoplasm)			1-3+ (occas)	1-3+ (occas)	Neg	Neg	Neg		Observed in spinal nerve roots.
Other elements			Neg	Neg	Neg	Neg	Neg		
**Spinal Cord**	****6-2	3,4						Pos	
Extracellular material			1-2+ (occas)	1-2+ (occas)	Neg	Neg	Neg		Observed in interstitium/stroma particularly in perivascular areas. Staining often appeared as diffuse to finely punctate material and often lined up on extracellular matrix fibers. Primarily observed in meninges.
Glial cells/processes (cytoplasm)			1-2+ (occas to freq)	1-2+ (occas)	Neg	Neg	Neg		
Axons (cytoplasm)			2-3+ (occas to freq)	2-3+ (occas)	Neg	Neg	Neg		
Cells/processes associated with peripheral nerves (cytoplasm)			1-3+ (occas)	1-3+ (occas)	Neg	Neg	Neg		Observed in spinal nerve roots.
Other elements			Neg	Neg	Neg	Neg	Neg		
**Spinal Cord**	***40-4	3,4						Pos	
Extracellular material			1-2+ (occas)	1-2+ (occas)	Neg	Neg	Neg		Observed in interstitium/stroma particularly in perivascular areas. Staining often appeared as diffuse to finely punctate material and often lined up on extracellular matrix fibers. Primarily observed in meninges.
Glial cells/processes (cytoplasm)			1-2+ (occas to freq)	1-2+ (occas)	Neg	Neg	Neg		

± = equivocal, 1+ = weak, 2+ = moderate, 3+ = strong, 4+ = intense, Neg = Negative, Pos = Positive, M = Missing, NE = Not Evaluated, NS = Not Stained, freq = frequent, occas = occasional. Frequency modifiers were included to provide the approximate percentage staining of expected numbers of that cell type or tissue element at that location. The frequency of cells with staining was identified as follows: very rare (<1% of cells of a particular cell type); rare (1-5% of cells of a particular cell type); rare to occasional (>5-25% of cells of a particular cell type); occasional (>25-50% of cells of a particular type); occasional to frequent (>50-75% of cells of a particular cell type); frequent (>75-100% of cells of a particular cell type).

S1 Table. Immunopathology Evaluation: Cross Reactivity of hResistin IgG with Normal Human Tissues

Tissue	Source	Run	Test Article (hResistin IgG) 20 µg/mL	Test Article (hResistin IgG) 5 µg/mL	Control Article (HuIgG1) 20 µg/mL	Control Article (HuIgG1) 5 µg/mL	Assay Control	Tissue Validation (Tissue Staining) Control	Tissue Comments/Nonspecific Findings
Axons (cytoplasm)			2-3+ (occas)	1-2+ (rare to occas)	Neg	Neg	Neg		
Cells/processes associated with peripheral nerves (cytoplasm)			1-3+ (occas)	1-3+ (occas)	Neg	Neg	Neg		Observed in spinal nerve roots.
Other elements			Neg	Neg	Neg	Neg	Neg		
**Spleen**	****7-9	3,4						Pos	
Extracellular material			1-3+ (occas to freq)	1-3+ (occas to freq)	Neg	Neg	Neg		Observed in interstitium/stroma particularly in perivascular areas. Staining often appeared as diffuse to finely punctate material and often lined up on extracellular matrix fibers.
Other elements			Neg	Neg	Neg	Neg	Neg		
**Spleen**	****28-5	3,4						Pos	
Extracellular material			1-3+ (occas)	1-3+ (occas)	Neg	Neg	Neg		Observed in interstitium/stroma particularly in perivascular areas. Staining often appeared as diffuse to finely punctate material and often lined up on extracellular matrix fibers.
Macrophages (cytoplasmic granules)			1-2+ (very rare)	1-2+ (very rare)	Neg	Neg	Neg		Primarily observed in germinal centers.
Other elements			Neg	Neg	Neg	Neg	Neg		

± = equivocal, 1+ = weak, 2+ = moderate, 3+ = strong, 4+ = intense, Neg = Negative, Pos = Positive, M = Missing, NE = Not Evaluated, NS = Not Stained, freq = frequent, occas = occasional. Frequency modifiers were included to provide the approximate percentage staining of expected numbers of that cell type or tissue element at that location. The frequency of cells with staining was identified as follows: very rare (<1% of cells of a particular cell type); rare (1-5% of cells of a particular cell type); rare to occasional (>5-25% of cells of a particular cell type); occasional (>25-50% of cells of a particular type); occasional to frequent (>50-75% of cells of a particular cell type); frequent (>75-100% of cells of a particular cell type).

S1 Table. Immunopathology Evaluation: Cross Reactivity of hResistin IgG with Normal Human Tissues

Tissue	Source	Run	Test Article (hResistin IgG)		Control Article (HuIgG1)		Assay Control	Tissue Validation (Tissue Staining) Control	Tissue Comments/Nonspecific Findings
			20 µg/mL	5 µg/mL	20 µg/mL	5 µg/mL			
**Spleen**	****10-5	3,4						Pos	
Extracellular material			1-3+ (occas to freq)	1-3+ (occas to freq)	Neg	Neg	Neg		Observed in interstitium/stroma particularly in perivascular areas. Staining often appeared as diffuse to finely punctate material and often lined up on extracellular matrix fibers.
Other elements			Neg	Neg	Neg	Neg	Neg		
**Striated Muscle (skeletal)**	****75-1	2,4						Pos	
Extracellular material			1-3+ (occas)	1-3+ (occas)	Neg	Neg	Neg		Observed in interstitium/stroma particularly in perivascular areas. Staining often appeared as diffuse to finely punctate material and often lined up on extracellular matrix fibers.
Other elements			Neg	Neg	Neg	Neg	Neg		
**Striated Muscle (skeletal)**	****09-4	2,4						Pos	
Extracellular material			1-3+ (occas)	1-3+ (occas)	Neg	Neg	Neg		Observed in interstitium/stroma particularly in perivascular areas. Staining often appeared as diffuse to finely punctate material and often lined up on extracellular matrix fibers.
Macrophages (cytoplasmic granules)			1-3+ (very rare)	1-3+ (very rare)	Neg	Neg	Neg		Observed scattered in interstitium.
Other elements			Neg	Neg	Neg	Neg	Neg		

± = equivocal, 1+ = weak, 2+ = moderate, 3+ = strong, 4+ = intense, Neg = Negative, Pos = Positive, M = Missing, NE = Not Evaluated, NS = Not Stained, freq = frequent, occas = occasional. Frequency modifiers were included to provide the approximate percentage staining of expected numbers of that cell type or tissue element at that location. The frequency of cells with staining was identified as follows: very rare (<1% of cells of a particular cell type); rare (1-5% of cells of a particular cell type); rare to occasional (>5-25% of cells of a particular cell type); occasional (>25-50% of cells of a particular type); occasional to frequent (>50-75% of cells of a particular cell type); frequent (>75-100% of cells of a particular cell type).

S1 Table. Immunopathology Evaluation: Cross Reactivity of hResistin IgG with Normal Human Tissues

Tissue	Source	Run	Test Article (hResistin IgG)		Control Article (HuIgG1)		Assay Control	Tissue Validation (Tissue Staining) Control	Tissue Comments/Nonspecific Findings
			20 µg/mL	5 µg/mL	20 µg/mL	5 µg/mL			
**Striated Muscle (skeletal)**	****63-1	2,4						Pos	
Extracellular material			1-3+ (occas)	1-3+ (occas)	Neg	Neg	Neg		Observed in interstitium/stroma particularly in perivascular areas. Staining often appeared as diffuse to finely punctate material and often lined up on extracellular matrix fibers.
Other elements			Neg	Neg	Neg	Neg	Neg		
**Testis**	***70-2	3,4						Pos	
Extracellular material			1-3+ (occas to freq)	1-3+ (occas to freq)	Neg	Neg	Neg		Observed in interstitium/stroma particularly in perivascular areas. Staining often appeared as diffuse to finely punctate material and often lined up on extracellular matrix fibers.
Other elements			Neg	Neg	Neg	Neg	Neg		
**Testis**	****18-1	3,4						Pos	
Extracellular material			1-3+ (occas to freq)	1-3+ (occas to freq)	Neg	Neg	Neg		Observed in interstitium/stroma particularly in perivascular areas. Staining often appeared as diffuse to finely punctate material and often lined up on extracellular matrix fibers.
Macrophages (cytoplasmic granules)			1-3+ (rare)	1-3+ (rare)	Neg	Neg	Neg		Observed scattered in interstitium.
Mesothelium (cytoplasm, cytoplasmic granules)			2-3+ (freq)	2-3+ (freq)	Neg	Neg	Neg		
Cells/processes associated with peripheral nerves (cytoplasm)			1-3+ (rare)	1-3+ (rare)	Neg	Neg	Neg		
Other elements			Neg	Neg	Neg	Neg	Neg		

± = equivocal, 1+ = weak, 2+ = moderate, 3+ = strong, 4+ = intense, Neg = Negative, Pos = Positive, M = Missing, NE = Not Evaluated, NS = Not Stained, freq = frequent, occas = occasional. Frequency modifiers were included to provide the approximate percentage staining of expected numbers of that cell type or tissue element at that location. The frequency of cells with staining was identified as follows: very rare (<1% of cells of a particular cell type); rare (1-5% of cells of a particular cell type); rare to occasional (>5-25% of cells of a particular cell type); occasional (>25-50% of cells of a particular type); occasional to frequent (>50-75% of cells of a particular cell type); frequent (>75-100% of cells of a particular cell type).

S1 Table. Immunopathology Evaluation: Cross Reactivity of hResistin IgG with Normal Human Tissues

Tissue	Source	Run	Test Article (hResistin IgG)		Control Article (HuIgG1)		Assay Control	Tissue Validation (Tissue Staining) Control	Tissue Comments/Nonspecific Findings
			20 µg/mL	5 µg/mL	20 µg/mL	5 µg/mL			
**Testis**	****81-1	3,4						Pos	
Extracellular material			1-3+ (occas to freq)	1-3+ (occas to freq)	Neg	Neg	Neg		Observed in interstitium/stroma particularly in perivascular areas. Staining often appeared as diffuse to finely punctate material and often lined up on extracellular matrix fibers.
Macrophages (cytoplasmic granules)			1-3+ (rare)	1-3+ (rare)	Neg	Neg	Neg		Observed scattered in interstitium.
Other elements			Neg	Neg	Neg	Neg	Neg		
**Thymus**	****04-2	3,4						Pos	
Extracellular material			1-2+ (occas to freq)	1-2+ (occas to freq)	Neg	Neg	Neg		Observed in interstitium/stroma particularly in perivascular areas. Staining often appeared as diffuse to finely punctate material and often lined up on extracellular matrix fibers.
Macrophages (cytoplasmic granules)			1-3+ (rare)	1-3+ (rare)	Neg	Neg	Neg		Observed scattered in interstitium.
Other elements			Neg	Neg	Neg	Neg	Neg		
**Thymus**	****05-2	3,4						Pos	
Extracellular material			1-2+ (occas to freq)	1-2+ (occas to freq)	Neg	Neg	Neg		Observed in interstitium/stroma particularly in perivascular areas. Staining often appeared as diffuse to finely punctate material and often lined up on extracellular matrix fibers.
Macrophages (cytoplasmic granules)			1-3+ (rare)	1-3+ (rare)	Neg	Neg	Neg		Observed scattered in interstitium.
Other elements			Neg	Neg	Neg	Neg	Neg		

± = equivocal, 1+ = weak, 2+ = moderate, 3+ = strong, 4+ = intense, Neg = Negative, Pos = Positive, M = Missing, NE = Not Evaluated, NS = Not Stained, freq = frequent, occas = occasional. Frequency modifiers were included to provide the approximate percentage staining of expected numbers of that cell type or tissue element at that location. The frequency of cells with staining was identified as follows: very rare (<1% of cells of a particular cell type); rare (1-5% of cells of a particular cell type); rare to occasional (>5-25% of cells of a particular cell type); occasional (>25-50% of cells of a particular type); occasional to frequent (>50-75% of cells of a particular cell type); frequent (>75-100% of cells of a particular cell type).

S1 Table. Immunopathology Evaluation: Cross Reactivity of hResistin IgG with Normal Human Tissues

Tissue	Source	Run	Test Article (hResistin IgG)		Control Article (HuIgG1)		Assay Control	Tissue Validation (Tissue Staining) Control	Tissue Comments/Nonspecific Findings
			20 µg/mL	5 µg/mL	20 µg/mL	5 µg/mL			
**Thymus**	****95-4	3,4						Pos	
Extracellular material			1-2+ (occas to freq)	1-2+ (occas to freq)	Neg	Neg	Neg		Observed in interstitium/stroma particularly in perivascular areas. Staining often appeared as diffuse to finely punctate material and often lined up on extracellular matrix fibers.
Macrophages (cytoplasmic granules)			1-3+ (rare)	1-3+ (rare)	Neg	Neg	Neg		Observed scattered in interstitium.
Other elements			Neg	Neg	Neg	Neg	Neg		
**Thyroid**	***80-11	3,4						Pos	
Extracellular material			1-2+ (occas to freq)	1-2+ (occas to freq)	Neg	Neg	Neg		Observed in interstitium/stroma particularly in perivascular areas. Staining often appeared as diffuse to finely punctate material and often lined up on extracellular matrix fibers. Also included colloid.
Other elements			Neg	Neg	Neg	Neg	Neg		
**Thyroid**	***78-2	3,4						Pos	
Extracellular material			1-2+ (occas to freq)	1-2+ (occas to freq)	Neg	Neg	Neg		Observed in interstitium/stroma particularly in perivascular areas. Staining often appeared as diffuse to finely punctate material and often lined up on extracellular matrix fibers. Also included colloid.
Other elements			Neg	Neg	Neg	Neg	Neg		

± = equivocal, 1+ = weak, 2+ = moderate, 3+ = strong, 4+ = intense, Neg = Negative, Pos = Positive, M = Missing, NE = Not Evaluated, NS = Not Stained, freq = frequent, occas = occasional. Frequency modifiers were included to provide the approximate percentage staining of expected numbers of that cell type or tissue element at that location. The frequency of cells with staining was identified as follows: very rare (<1% of cells of a particular cell type); rare (1-5% of cells of a particular cell type); rare to occasional (>5-25% of cells of a particular cell type); occasional (>25-50% of cells of a particular type); occasional to frequent (>50-75% of cells of a particular cell type); frequent (>75-100% of cells of a particular cell type).

S1 Table. Immunopathology Evaluation: Cross Reactivity of hResistin IgG with Normal Human Tissues

Tissue	Source	Run	Test Article (hResistin IgG)		Control Article (HuIgG1)		Assay Control	Tissue Validation (Tissue Staining) Control	Tissue Comments/Nonspecific Findings
			20 µg/mL	5 µg/mL	20 µg/mL	5 µg/mL			
**Thyroid**	***85	3,4						Pos	
Extracellular material			1-2+ (occas to freq)	1-2+ (occas to freq)	Neg	Neg	Neg		Observed in interstitium/stroma particularly in perivascular areas. Staining often appeared as diffuse to finely punctate material and often lined up on extracellular matrix fibers. Also included colloid.
Other elements			Neg	Neg	Neg	Neg	Neg		
**Tonsil**	****10-2	3,4						Pos	
Extracellular material			1-3+ (occas)	1-3+ (occas)	Neg	Neg	Neg		Observed in interstitium/stroma particularly in perivascular areas. Staining often appeared as diffuse to finely punctate material and often lined up on extracellular matrix fibers.
Macrophages (cytoplasmic granules)			1-2+ (rare)	1-2+ (rare)	Neg	Neg	Neg		Primarily observed in germinal centers.
Other elements			Neg	Neg	Neg	Neg	Neg		
**Tonsil**	****59-1	3,4						Pos	
Extracellular material			1-2+ (occas)	1-2+ (occas)	Neg	Neg	Neg		Observed in interstitium/stroma particularly in perivascular areas. Staining often appeared as diffuse to finely punctate material and often lined up on extracellular matrix fibers.
Macrophages (cytoplasmic granules)			1-2+ (rare)	1-2+ (rare)	Neg	Neg	Neg		Primarily observed in germinal centers.
Other elements			Neg	Neg	Neg	Neg	Neg		

± = equivocal, 1+ = weak, 2+ = moderate, 3+ = strong, 4+ = intense, Neg = Negative, Pos = Positive, M = Missing, NE = Not Evaluated, NS = Not Stained, freq = frequent, occas = occasional. Frequency modifiers were included to provide the approximate percentage staining of expected numbers of that cell type or tissue element at that location. The frequency of cells with staining was identified as follows: very rare (<1% of cells of a particular cell type); rare (1-5% of cells of a particular cell type); rare to occasional (>5-25% of cells of a particular cell type); occasional (>25-50% of cells of a particular type); occasional to frequent (>50-75% of cells of a particular cell type); frequent (>75-100% of cells of a particular cell type).

S1 Table. Immunopathology Evaluation: Cross Reactivity of hResistin IgG with Normal Human Tissues

Tissue	Source	Run	Test Article (hResistin IgG)		Control Article (HuIgG1)		Assay Control	Tissue Validation (Tissue Staining) Control	Tissue Comments/Nonspecific Findings
			20 µg/mL	5 µg/mL	20 µg/mL	5 µg/mL			
**Tonsil**	****56-2	3,4						Pos	
Extracellular material			1-2+ (occas)	1-2+ (occas)	Neg	Neg	Neg		Observed in interstitium/stroma particularly in perivascular areas. Staining often appeared as diffuse to finely punctate material and often lined up on extracellular matrix fibers.
Macrophages (cytoplasmic granules)			1-2+ (rare)	1-2+ (rare)	Neg	Neg	Neg		Primarily observed in germinal centers.
Other elements			Neg	Neg	Neg	Neg	Neg		
**Ureter**	****89-1	3,4						Pos	
Extracellular material			1-3+ (occas to freq)	1-3+ (occas to freq)	Neg	Neg	Neg		Observed in interstitium/stroma particularly in perivascular areas. Staining often appeared as diffuse to finely punctate material and often lined up on extracellular matrix fibers.
Macrophages (cytoplasmic granules)			1-3+ (occas)	1-3+ (occas)	Neg	Neg	Neg		Observed scattered in interstitium.
Other elements			Neg	Neg	Neg	Neg	Neg		
**Ureter**	****84-2	3,4						Pos	
Extracellular material			1-2+ (occas to freq)	1-2+ (occas to freq)	Neg	Neg	Neg		Observed in interstitium/stroma particularly in perivascular areas. Staining often appeared as diffuse to finely punctate material and often lined up on extracellular matrix fibers.
Macrophages (cytoplasmic granules)			1-3+ (rare)	1-3+ (rare)	Neg	Neg	Neg		Observed scattered in interstitium.
Other elements			Neg	Neg	Neg	Neg	Neg		

± = equivocal, 1+ = weak, 2+ = moderate, 3+ = strong, 4+ = intense, Neg = Negative, Pos = Positive, M = Missing, NE = Not Evaluated, NS = Not Stained, freq = frequent, occas = occasional. Frequency modifiers were included to provide the approximate percentage staining of expected numbers of that cell type or tissue element at that location. The frequency of cells with staining was identified as follows: very rare (<1% of cells of a particular cell type); rare (1-5% of cells of a particular cell type); rare to occasional (>5-25% of cells of a particular cell type); occasional (>25-50% of cells of a particular type); occasional to frequent (>50-75% of cells of a particular cell type); frequent (>75-100% of cells of a particular cell type).

S1 Table. Immunopathology Evaluation: Cross Reactivity of hResistin IgG with Normal Human Tissues

Tissue	Source	Run	Test Article (hResistin IgG)		Control Article (HuIgG1)		Assay Control	Tissue Validation (Tissue Staining) Control	Tissue Comments/Nonspecific Findings
			20 µg/mL	5 µg/mL	20 µg/mL	5 µg/mL			
**Ureter**	****91-2	3,4						Pos	Exogenous pigment from tissue marking ink.
Extracellular material			1-3+ (occas to freq)	1-3+ (occas to freq)	Neg	Neg	Neg		Observed in interstitium/stroma particularly in perivascular areas. Staining often appeared as diffuse to finely punctate material and often lined up on extracellular matrix fibers.
Macrophages (cytoplasmic granules)			1-3+ (occas)	1-3+ (occas)	Neg	Neg	Neg		Observed scattered in interstitium.
Other elements			Neg	Neg	Neg	Neg	Neg		
**Uterus – cervix**	****51-1	1,4						Pos	External ostium.
Extracellular material			1-3+ (occas to freq)	1-3+ (occas to freq)	Neg	Neg	Neg		Observed in interstitium/stroma particularly in perivascular areas. Staining often appeared as diffuse to finely punctate material and often lined up on extracellular matrix fibers.
Macrophages (cytoplasmic granules)			1-3+ (very rare)	1-3+ (very rare)	Neg	Neg	Neg		Observed scattered in interstitium.
Other elements			Neg	Neg	Neg	Neg	Neg		
**Uterus – cervix**	****57-1	1,4						Pos	External ostium.
Extracellular material			1-3+ (occas to freq)	1-3+ (occas to freq)	Neg	Neg	Neg		Observed in interstitium/stroma particularly in perivascular areas. Staining often appeared as diffuse to finely punctate material and often lined up on extracellular matrix fibers.
Macrophages (cytoplasmic granules)			1-3+ (very rare)	1-3+ (very rare)	Neg	Neg	Neg		Observed scattered in interstitium.
Other elements			Neg	Neg	Neg	Neg	Neg		

± = equivocal, 1+ = weak, 2+ = moderate, 3+ = strong, 4+ = intense, Neg = Negative, Pos = Positive, M = Missing, NE = Not Evaluated, NS = Not Stained, freq = frequent, occas = occasional. Frequency modifiers were included to provide the approximate percentage staining of expected numbers of that cell type or tissue element at that location. The frequency of cells with staining was identified as follows: very rare (<1% of cells of a particular cell type); rare (1-5% of cells of a particular cell type); rare to occasional (>5-25% of cells of a particular cell type); occasional (>25-50% of cells of a particular type); occasional to frequent (>50-75% of cells of a particular cell type); frequent (>75-100% of cells of a particular cell type).

S1 Table. Immunopathology Evaluation: Cross Reactivity of hResistin IgG with Normal Human Tissues

Tissue	Source	Run	Test Article (hResistin IgG)		Control Article (HuIgG1)		Assay Control	Tissue Validation (Tissue Staining) Control	Tissue Comments/Nonspecific Findings
			20 µg/mL	5 µg/mL	20 µg/mL	5 µg/mL			
**Uterus – cervix**	****12-4	1,4						Pos	External ostium.
Extracellular material			1-3+ (occas to freq)	1-3+ (occas to freq)	Neg	Neg	Neg		Observed in interstitium/stroma particularly in perivascular areas. Staining often appeared as diffuse to finely punctate material and often lined up on extracellular matrix fibers.
Macrophages (cytoplasmic granules)			1-3+ (very rare)	1-3+ (very rare)	Neg	Neg	Neg		Observed scattered in interstitium.
Other elements			Neg	Neg	Neg	Neg	Neg		
**Uterus – endometrium**	****92-12	3,4	NE	NE	NE	NE	NE	NE	Endometrium not present in sections. Tissue judged inadequate for evaluation and recorded as not evaluated (NE). Replaced with HT1093-7 in run 5.
**Uterus – endometrium**	****93-7	5,6						Pos	Replacement for HT1092-12.
Extracellular material			1-3+ (occas to freq)	1-3+ (occas to freq)	Neg	Neg	Neg		Observed in interstitium/stroma particularly in perivascular areas. Staining often appeared as diffuse to finely punctate material and often lined up on extracellular matrix fibers.
Macrophages (cytoplasmic granules)			1-3+ (rare)	1-3+ (rare)	Neg	Neg	Neg		Observed scattered in interstitium.
Other elements			Neg	Neg	Neg	Neg	Neg		
**Uterus – endometrium**	****11-2	3,4						Pos	
Extracellular material			1-3+ (occas to freq)	1-3+ (occas to freq)	Neg	Neg	Neg		Observed in interstitium/stroma particularly in perivascular areas. Staining often appeared as diffuse to finely punctate material and often lined up on extracellular matrix fibers.

± = equivocal, 1+ = weak, 2+ = moderate, 3+ = strong, 4+ = intense, Neg = Negative, Pos = Positive, M = Missing, NE = Not Evaluated, NS = Not Stained, freq = frequent, occas = occasional. Frequency modifiers were included to provide the approximate percentage staining of expected numbers of that cell type or tissue element at that location. The frequency of cells with staining was identified as follows: very rare (<1% of cells of a particular cell type); rare (1-5% of cells of a particular cell type); rare to occasional (>5-25% of cells of a particular cell type); occasional (>25-50% of cells of a particular type); occasional to frequent (>50-75% of cells of a particular cell type); frequent (>75-100% of cells of a particular cell type).

S1 Table. Immunopathology Evaluation: Cross Reactivity of hResistin IgG with Normal Human Tissues

Tissue	Source	Run	Test Article (hResistin IgG) 20 µg/mL	Test Article (hResistin IgG) 5 µg/mL	Control Article (HuIgG1) 20 µg/mL	Control Article (HuIgG1) 5 µg/mL	Assay Control	Tissue Validation (Tissue Staining) Control	Tissue Comments/Nonspecific Findings
Macrophages (cytoplasmic granules)			1-3+ (very rare)	1-3+ (very rare)	Neg	Neg	Neg		Observed scattered in interstitium.
Other elements			Neg	Neg	Neg	Neg	Neg		
**Uterus – endometrium**	****51-1	5,6						Pos	Replacement for HT2379-1.
Extracellular material			1-3+ (occas to freq)	1-3+ (occas to freq)	Neg	Neg	Neg		Observed in interstitium/stroma particularly in perivascular areas. Staining often appeared as diffuse to finely punctate material and often lined up on extracellular matrix fibers.
Macrophages (cytoplasmic granules)			1-3+ (very rare)	1-3+ (very rare)	Neg	Neg	Neg		Observed scattered in interstitium.
Other elements			Neg	Neg	Neg	Neg	Neg		
**Uterus – endometrium**	****79-1	3,4	NE	NE	NE	NE	NE	NE	Endometrium not present in sections. Tissue judged inadequate for evaluation and recorded as not evaluated (NE). Replaced with HT2251-1 in run 5.

± = equivocal, 1+ = weak, 2+ = moderate, 3+ = strong, 4+ = intense, Neg = Negative, Pos = Positive, M = Missing, NE = Not Evaluated, NS = Not Stained, freq = frequent, occas = occasional. Frequency modifiers were included to provide the approximate percentage staining of expected numbers of that cell type or tissue element at that location. The frequency of cells with staining was identified as follows: very rare (<1% of cells of a particular cell type); rare (1-5% of cells of a particular cell type); rare to occasional (>5-25% of cells of a particular cell type); occasional (>25-50% of cells of a particular type); occasional to frequent (>50-75% of cells of a particular cell type); frequent (>75-100% of cells of a particular cell type).
