# Supplementary material for: Systemic evaluation and localization of resistin expression in normal human tissues by a newly developed monoclonal antibody
Source: PLoS One. 2020 Jul 1;15(7):e0235546. doi: 10.1371/journal.pone.0235546 (PMC7329134; doi:10.1371/journal.pone.0235546)
Supplement: S1 Raw Images — (PDF) [file pone.0235546.s002.pdf]

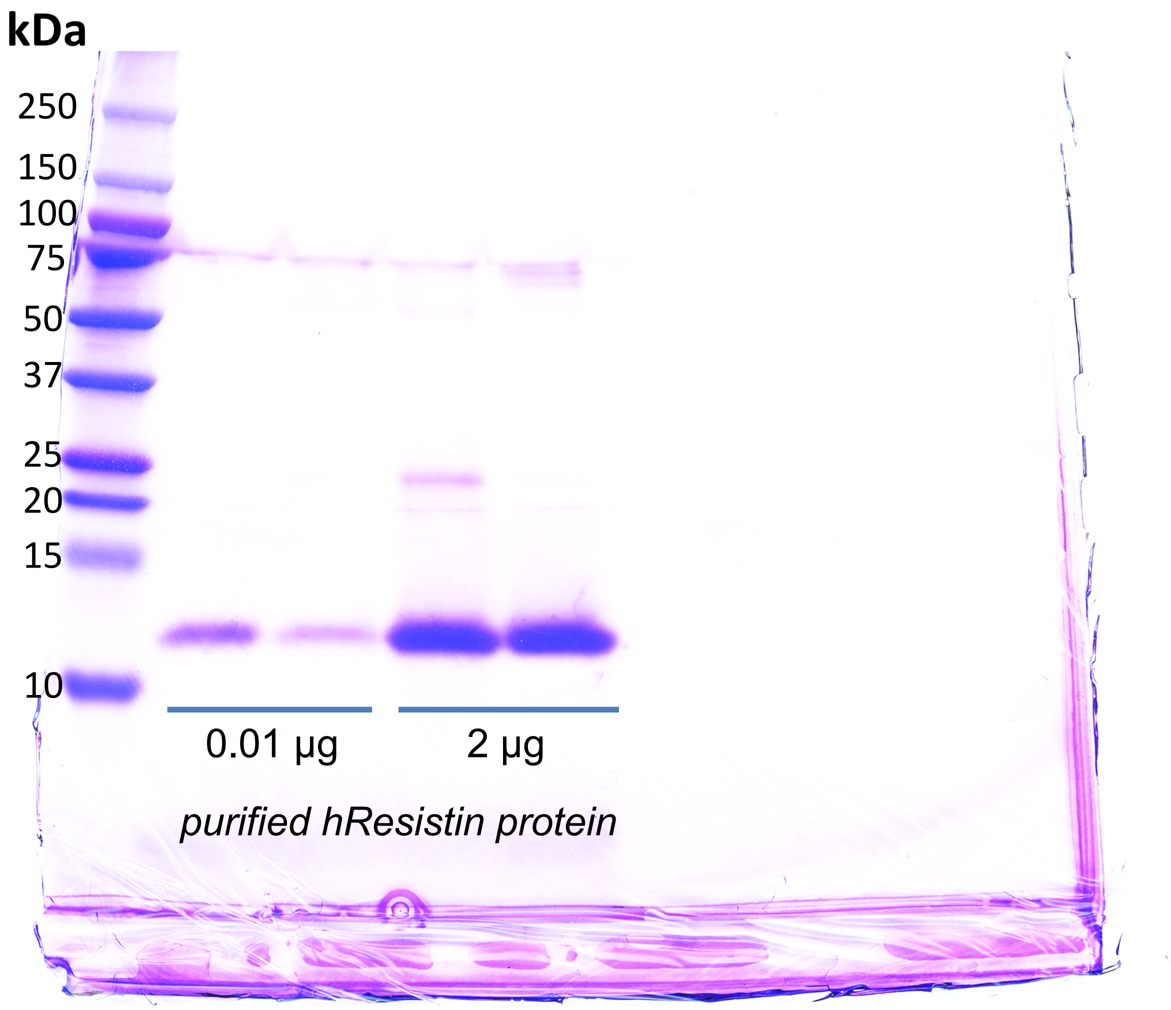

Fig 1. (**A**) Coomassie-stained gel shows SDS-PAGE analysis of purified recombinant hResistin protein. Gels were stained with Coomassie Brilliant Blue (#1610436, Bio-Rad) and the image was captured using an Epson Perfection scanner.

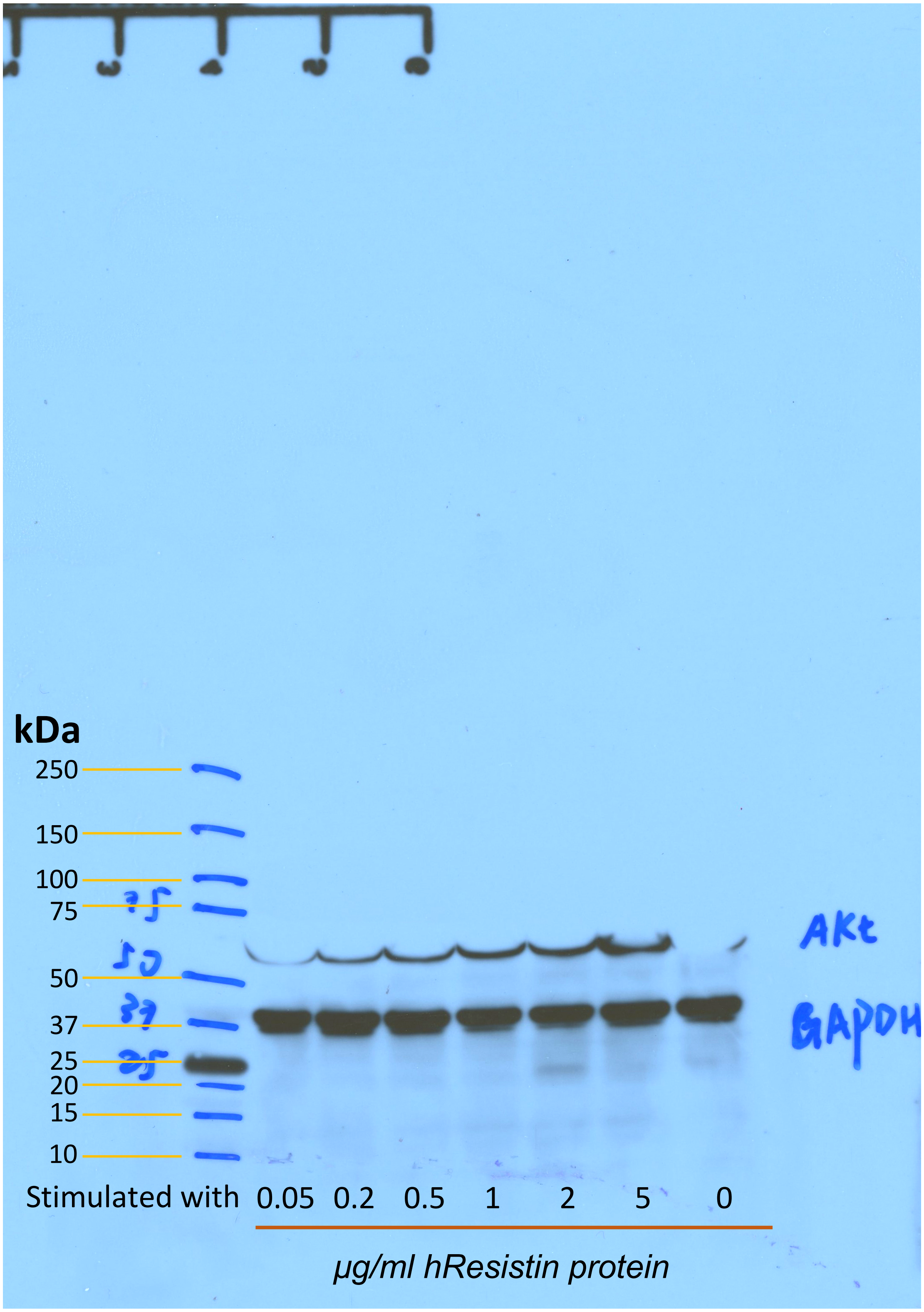

Fig 1. (**B**) Western blotting shows Akt phosphorylation induced by the purified hResistin proteins in the 3T3-L1 cell line. Protein bands were visualized by chemiluminescence (ECL; RPN2106, GE Healthcare) and exposure to the HyBlot CL® Autoradiography films (E3018, Denville Scientific Inc.). The image was captured using an Epson Perfection scanner.

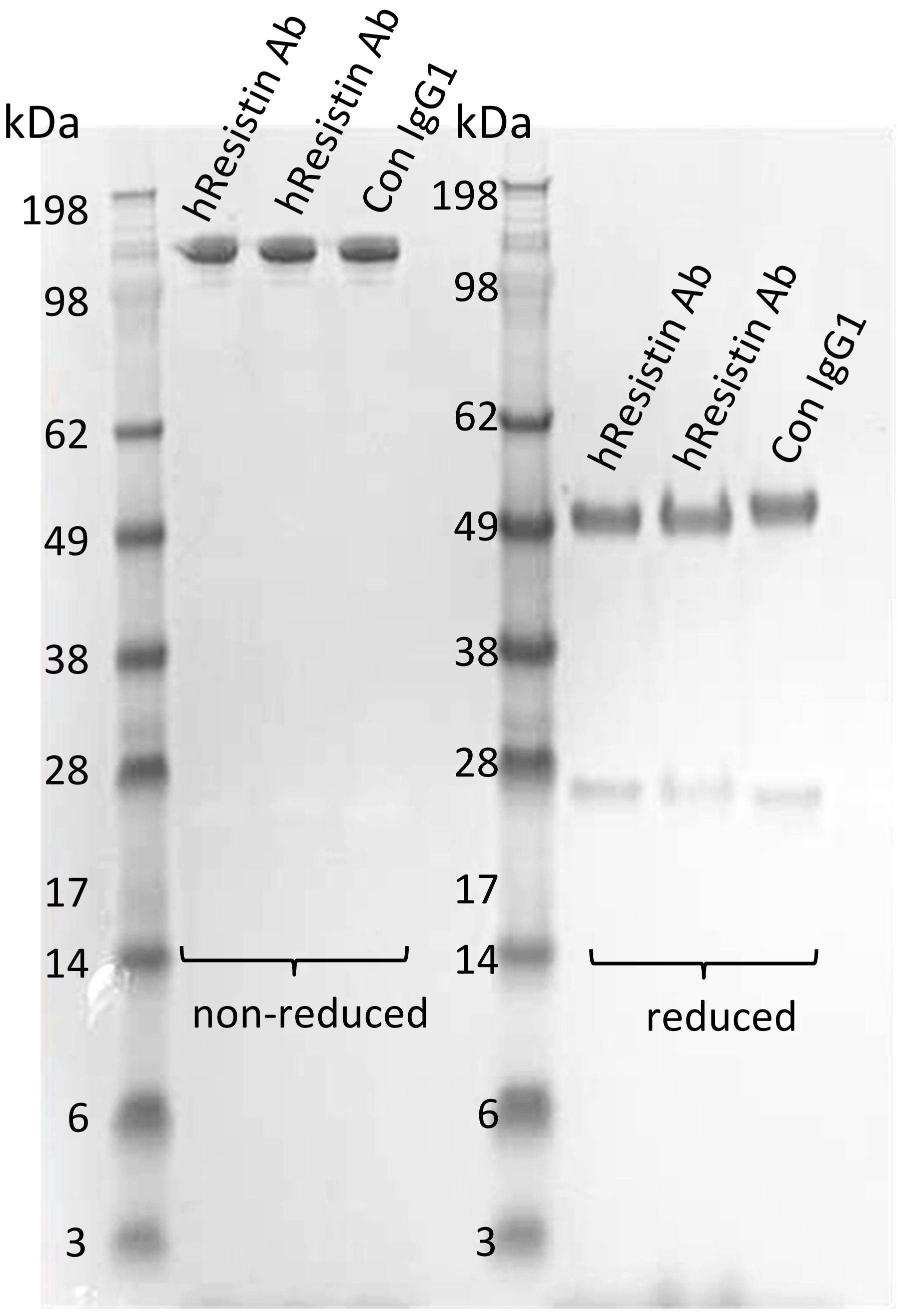

Fig 1. (**C**) Purity of the anti-hResistin antibody and the control human IgG1 were analyzed by SDS-PAGE. Gels were stained with InstantBlue (ISB1L, Sigma-Aldrich). The image was captured on a BioSpectrum Imaging System (UVP).
