# Supplementary material for: Systemic evaluation and localization of resistin expression in normal human tissues by a newly developed monoclonal antibody
Source: PLoS One. 2020 Jul 1;15(7):e0235546. doi: 10.1371/journal.pone.0235546 (PMC7329134; doi:10.1371/journal.pone.0235546)
Supplement: Attachment — Submitted filename: Response to Reviewers (May 21, 2020).docx [file pone.0235546.s003.docx]

**Point-by-point responses to:**

**Academic Editor’s Comments for Journal Requirements:**

*1. Please ensure that your manuscript meets PLOS ONE's style requirements, including those for file naming. The PLOS ONE style templates can be found at*

[*http://www.journals.plos.org/plosone/s/file?id=wjVg/PLOSOne_formatting_sample_main_body.pdf*](http://www.journals.plos.org/plosone/s/file?id=wjVg/PLOSOne_formatting_sample_main_body.pdf) *and* [*http://www.journals.plos.org/plosone/s/file?id=ba62/PLOSOne_formatting_sample_title_authors_affiliations.pdf*](http://www.journals.plos.org/plosone/s/file?id=ba62/PLOSOne_formatting_sample_title_authors_affiliations.pdf)

**Response:** We thank the editor for this reminder. With kind help from Ms. Claire Levine, MS, ELS, who is the scientific editor in our department (Anesthesiology and Critical Care Medicine, Johns Hopkins University), the manuscript has been formatted to meet *PLOS ONE*'s style requirements including the file naming.

*2. Thank you for including your ethics statement:*

*"Before the start of this research, the “Ethics Committee Approval for the Use of Human Tissues in Tissue Cross-Reactivity Studies” was obtained. Documentation of informed consent was required by this statement indicating that “informed consent shall be documented by the use of a written consent form approved by the IRB and signed by the subject or the subject's legally authorized representative, and a copy shall be given
to the person signing the form”.*

*Once you have amended this statement in the Methods section of the manuscript, please add the same text to the “Ethics Statement” field of the submission form (via “Edit Submission”).*

*For additional information about PLOS ONE ethical requirements for human subjects research, please refer to* [*http://journals.plos.org/plosone/s/submission-guidelines#loc-human-subjects-research*](http://journals.plos.org/plosone/s/submission-guidelines#loc-human-subjects-research)*.*

**Response:** We greatly appreciate the editor’s advice. We have amended the statement accordingly in the Methods section of the revised manuscript (pages 6: lines 94 to 99). We also made the same changes to “Ethics Statement” field of the submission form.

*3. We note that you have indicated that data from this study are available upon request. PLOS only allows data to be available upon request if there are legal or ethical restrictions on sharing data publicly. For information on unacceptable data access restrictions, please see* [*http://journals.plos.org/plosone/s/data-availability#loc-unacceptable-data-access-restrictions*](http://journals.plos.org/plosone/s/data-availability#loc-unacceptable-data-access-restrictions)*.*

*In your revised cover letter, please address the following prompts:*

*3-a) If there are ethical or legal restrictions on sharing a de-identified data set, please explain them in detail (e.g., data contain potentially identifying or sensitive patient information) and who has imposed them (e.g., an ethics committee). Please also provide contact information for a data access committee, ethics committee, or other institutional body to which data requests may be sent.*

*3-b) If there are no restrictions, please upload the minimal anonymized data set necessary to replicate your study findings as either Supporting Information files or to a stable, public repository and provide us with the relevant URLs, DOIs, or accession numbers. Please see http://www.bmj.com/content/340/bmj.c181.long for guidelines on how to de-identify and prepare clinical data for publication. For a list of acceptable repositories, please see* [*http://journals.plos.org/plosone/s/data-availability#loc-recommended-repositories*](http://journals.plos.org/plosone/s/data-availability#loc-recommended-repositories)*.*

*We will update your Data Availability statement on your behalf to reflect the information you provide.*

**Response:** We thank the editor for these instructions. We are sorry for any misunderstanding that our previous description might have caused, as actually all data underlying the findings had been presented in the originally submitted manuscript, which we believe met requirements of the PLOS-defined “minimal data set.” However, to further strengthen our findings, in the revised manuscript we provide the entire immunopathology evaluation used to determine cross reactivity of the anti-hResistin antibody with normal human tissues as supporting information (S1 Table). Now “all relevant data are within the revised paper and its supporting information files,” as we stated in the submission form and in revised manuscript (page 36). We also adjusted the related text description in the Methods section (page 11: lines 199 to 201) and addressed this concern in the revised cover letter.

*4. We note that you have included the phrase “data not shown” in your manuscript. Unfortunately, this does not meet our data sharing requirements. PLOS does not permit references to inaccessible data. We require that authors provide all relevant data within the paper, Supporting Information files, or in an acceptable, public repository. Please add a citation to support this phrase or upload the data that corresponds with these findings to a stable repository (such as Figshare or Dryad) and provide and URLs, DOIs, or accession numbers that may be used to access these data. Or, if the data are not a core part of the research being presented in your study, we ask that you remove the phrase that refers to these data.*

**Response:** The S1 Table provided as a Supporting Information file in the revised manuscript contains data to support these findings. We accordingly added a citation (S1 Table) to replace the “data not shown” in the revised manuscript (page 14: line 240).

*5. PLOS requires an ORCID iD for the corresponding author in Editorial Manager on papers submitted after December 6th, 2016. Please ensure that you have an ORCID iD and that it is validated in Editorial Manager. To do this, go to ‘Update my Information’ (in the upper left-hand corner of the main menu), and click on the Fetch/Validate link next to the ORCID field. This will take you to the ORCID site and allow you to create a new iD or authenticate a pre-existing iD in Editorial Manager. Please see the following video for instructions on linking an ORCID iD to your Editorial Manager account:* [*https://www.youtube.com/watch?v=_xcclfuvtxQ*](https://www.youtube.com/watch?v=_xcclfuvtxQ)

**Response:** The corresponding author, Dr. Roger A. Johns, has a pre-existing ORCID: “0000-0001-9232-2434”. It has been updated in the submission system.

*6. Thank you for stating the following in the Financial Disclosure section:*

*"This work was supported by National Institutes of Health (NIH) Centers for Advanced
Diagnostics and Experimental Therapeutics in Lung Diseases (CADET) Grant NHLBI
5UH2HL123827 (PI: R.A.J)."*

*We note that one or more of the authors are employed by a commercial company: "Charles River Laboratories, Inc."*

*6-a) Please provide an amended Funding Statement declaring this commercial affiliation, as well as a statement regarding the Role of Funders in your study. If the funding organization did not play a role in the study design, data collection and analysis, decision to publish, or preparation of the manuscript and only provided financial support in the form of authors' salaries and/or research materials, please review your statements relating to the author contributions, and ensure you have specifically and accurately indicated the role(s) that these authors had in your study. You can update author roles in the Author Contributions section of the online submission form.*

*Please also include the following statement within your amended Funding Statement.*

*“The funder provided support in the form of salaries for authors [insert relevant initials], but did not have any additional role in the study design, data collection and analysis, decision to publish, or preparation of the manuscript. The specific roles of these authors are articulated in the ‘author contributions’ section.”*

*If your commercial affiliation did play a role in your study, please state and explain this role within your updated Funding Statement.*

*6-b) Please also provide an updated Competing Interests Statement declaring this commercial affiliation along with any other relevant declarations relating to employment, consultancy, patents, products in development, or marketed products, etc.*

*Within your Competing Interests Statement, please confirm that this commercial affiliation does not alter your adherence to all PLOS ONE policies on sharing data and materials by including the following statement: "This does not alter our adherence to  PLOS ONE policies on sharing data and materials.” (as detailed online in our guide for authors* [*http://journals.plos.org/plosone/s/competing-interests*](http://journals.plos.org/plosone/s/competing-interests)*) . If this adherence statement is not accurate and  there are restrictions on sharing of data and/or materials, please state these. Please note that we cannot proceed with consideration of your article until this information has been declared.*

*Please include both an updated Funding Statement and Competing Interests Statement in your cover letter. We will change the online submission form on your behalf.*

*Please know it is PLOS ONE policy for corresponding authors to declare, on behalf of all authors, all potential competing interests for the purposes of transparency. PLOS defines a competing interest as anything that interferes with, or could reasonably be perceived as interfering with, the full and objective presentation, peer review, editorial decision-making, or publication of research or non-research articles submitted to one of the journals. Competing interests can be financial or non-financial, professional, or personal. Competing interests can arise in relationship to an organization or another person. Please follow this link to our website for more details on competing interests:* [*http://journals.plos.org/plosone/s/competing-interests*](http://journals.plos.org/plosone/s/competing-interests)

**Response:** We thank the editor for pointing out this matter. In the revised manuscript, we have amended the Funding Statement to clarify the role of Charles River Laboratories, Inc. in this study (page 35: lines 558 to 562). Charles River Laboratories, Inc. is not a funder. It is a contract research organization that performed the immunopathological study for determining the cross reactivity of anti-hResistin antibody in normal human tissues. The co-author Dr. Shari A. Price is an employee of Charles River Laboratories, Inc. The specific roles of these authors are articulated in the “author contributions” section (page 37) in the revised manuscript.

As advised, the Competing Interests Statement also has been updated in the revised manuscript (page 36). In addition to the clarification of the role of Charles River Laboratories, Inc., we also declared that Dr. Roger A. Johns has US (US 2016/0130341 A1) and international (WO 2014/204941 A1) patent applications pending for the monoclonal antibody developed against human resistin to cover pulmonary, cardiac, and other related inflammatory disorders. We confirmed that “This does not alter our adherence to *PLOS ONE* policies on sharing data and materials” at the end of the Competing Interests Statement.

Both the updated Funding Statement and Competing Interests Statement have been included in the revised cover letter. We understand that all of these amendments are required by *PLOS ONE* policy.

**Point-by-point responses to:**

**Reviewer’s Comments:**

Reviewer #1: *Thank you for inviting me to review this manuscript. The authors are to be commended on performing a rigorous study of the distribution of human resistin in human tissues and for presenting the results in an intelligible and well-organized fashion. The manuscript is sound. I have a couple of minor comments, relating to the methodology:*

**Response:** We greatly appreciate the reviewer’s general comments, and have addressed specific comments in a point-by-point manner as follows.

*-the authors should explain why they selected the antibodies used for testing (p4, l.65-67), or provide relevant references.*

**Response:** In our previous studies, we showed that the resistin-like molecule (RELM) α exhibited pro-proliferative effects through Akt phosphorylation on rodent pulmonary smooth muscle cells (*Circ Res. 2003, PMID: 12714564*) and human mesenchymal stem cells (*Stem Cells Dev. 2013, PMID: 22891677*). Similarly we had found that both RELMα and its human homolog resistin (hResistin) also could induce Akt phosphorylation in mouse embryonic fibroblast (adipocyte precursor) 3T3-L1 cells. Thus Akt phosphorylation has been employed as an indicator for validating the functional activities of RELMα and hResistin that were produced and purified by our laboratory (*J Immunol. 2019, PMID: 31611261*). Relevant references are provided in the revised manuscript to support the methodology (page 5: line 70; references: 2, 18 and 20).

*-the authors should explain the non-linear grading system used in table 4 (i.e. frequencies of <25% can fall within one of 3 frequency categories - very rare, rare or rare to occasional - whereas each subsequent frequency is a denomination of 25%. How and why did they select the cutoffs? Is there a supporting reference?*

**Response:** We thank the reviewer for pointing out this matter. The staining frequency scales in Table 4 were based on the literature-reported IHC quantitative approaches and resistin biology. Human resistin is mainly produced by macrophages (*BBRC. 2003, PMID: 12504108*), and as a secretory cytokine, it has both intracellular and extracellular localization. Thus, we specifically referred to the established IHC scoring systems used to analyze targeted proteins with a similar expression pattern. The scores dichotomized at the 25% cutoff point as rare vs. variable/occasional are well-accepted for measuring the percent of cells and area positively stained by IHC (*Diagn Pathol. 2014, PMID: 25432701; J Biomed Mater Res A. 2014, PMID: 23765602; PLoS One. 2014, PMID: 24802416*). Those tissue elements with >25% positive resistin staining were proposed to comprise the majority of extracellularly secreted protein, whereas those with <25% positive levels were considered to have predominately intracellular expression (*J Biomed Mater Res A. 2014, PMID: 23765602*). In the above-listed references, sample denominations of 25% (i.e., 50%, 75%, and 100) were used for staining with >25% frequency. For those tissues with a rare level of resistin signal (<25%), we chose 1% and 5% as cut-points based on the established quantification specific for intracellular staining evaluation (*Diagn Pathol. 2014, PMID: 25432701; J Clin Pathol. 1995, PMID: 7490328; Mod Pathol. 1998, PMID: 9504686*). It has been difficult to find published data on the ratio of resistin-positive cells/total cells in normal human tissues. As a human homolog of rodent RELMα and an M2 macrophage marker (as discussed in page 3, line 33 with reference-10 in the revised manuscript), human resistin is likely also expressed by M2-like macrophage subsets. According to the literature, in virtually all adult mammal tissues, resident macrophages could represent up to 10% of the total cell number in quiescent conditions (*Front Immunol. 2014, PMID: 25368618*). The ratio of human M1 and M2 macrophages remains in a 1:1 balance under normal conditions (*Exp Ther Med. 2018, PMID: 30546406*). Therefore, the resistin-positive cells are likely less than 5% in normal human tissues. The resistin expression in human lung could serve as a clue because it has been implicated in pulmonary diseases (*J Immunol. 2019, PMID: 31611261*). In noncancerous transplanted human lungs *(Sci Transl Med. 2019, PMID: 30760579*), the M2-like macrophage frequency among all nucleated cells was approximately 1% to 5%, which was concordant with the detected resistin-positive macrophage-like cells in human transplanted lungs without pulmonary hypertension in our previous study (*J Immunol. 2019, PMID: 31611261*). Moreover, we originally had planned that these proposed scales might be modified at our expert pathologists’ discretion to better reflect the staining frequency seen during evaluation. Subsequently, the results (presented in this manuscript) turned out to strengthen the evidence for using this nonlinear grading system as an appropriate tool for resistin expression analysis. Collectively, we believe that this information could theoretically and practically support the proposed cutoffs set up as the scales in Table 4. In the revised manuscript, we added a brief discussion to clarify the rationale for this IHC quantitative approach (page 11: lines 194 to 198). Related supporting references (# 24-30) are also provided.
